# Supplementary material for: Improving recombinant protein production by yeast through genome-scale modeling using proteome constraints
Source: Nat Commun. 2022 May 27;13:2969. doi: 10.1038/s41467-022-30689-7 (PMC9142503; doi:10.1038/s41467-022-30689-7)
Supplement: Supplementary file 1 — Supplementary Information [file 41467_2022_30689_MOESM1_ESM.pdf]

Improving recombinant protein production by yeast through genome-scale modeling using proteome constraints

Li *et al.*

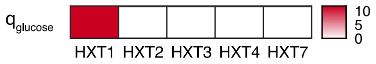

Supplementary Figure 1. Fluxes carried by glucose transporters at maximum growth simulation when both Hxt1 and Hxt7 were set with the k_{cat} value for Hxt7 (197 s^{-1}). Source data are provided as a Source Data file.

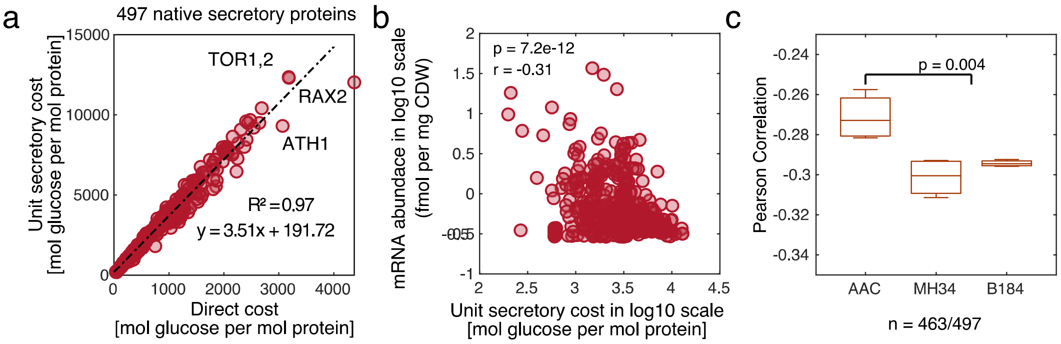

Supplementary Figure 2. *S. cerevisiae* suppress costly native proteins upon recombinant protein production. a) Simulated secretory costs for 497 native secretory proteins in *S. cerevisiae*. Direct cost includes the energetic cost for synthesis, modification and secretion of this protein, while unit secretory cost additionally includes the cost for the corresponding increased fraction of the catalytic machineries in these processes caused by the increase of this protein. b) Example of negative Pearson correlation of mRNA levels with unit secretory costs of native secretory and cell membrane proteins in MH34 strain at 0.1 h^{-1}. Student's t test was used to calculate *P* value for Pearson's correlation. c) Pearson correlation of mRNA level with glucose cost of native secretory proteins for three α-amylase strains. *P* value for the AAC with the MH34 and B184 was calculated using two-sided Wilcoxon rank sum test. In the boxplot, the central band represents median value, box represents the upper and lower quartiles, and the whiskers extend up to 1.5 times the interquartile range beyond the box range. AAC: low yield α-amylase strain; MH34 and B184: high yield α-amylase strain. 463 out of 497 native proteins have measured mRNA abundances and were used in the correlation analysis. Each point in the box plot represents one correlation and has *P* value < 1e-8. Source data are provided as a Source Data file.

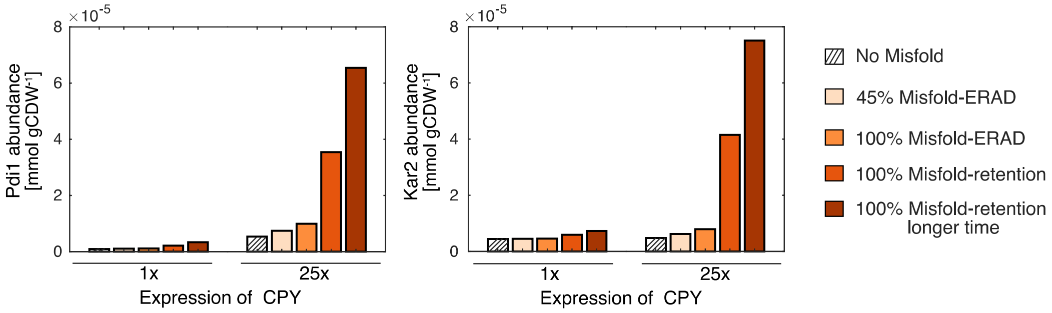

Supplementary Figure 3. Simulated abundances of Kar2 and Pdi1 in expression of different amount of CPY with different routes. 1x means its native expression level, 25x means 25-fold of its native expression level. Native expression level of CPY is from PaxDb database. Source data are provided as a Source Data file.

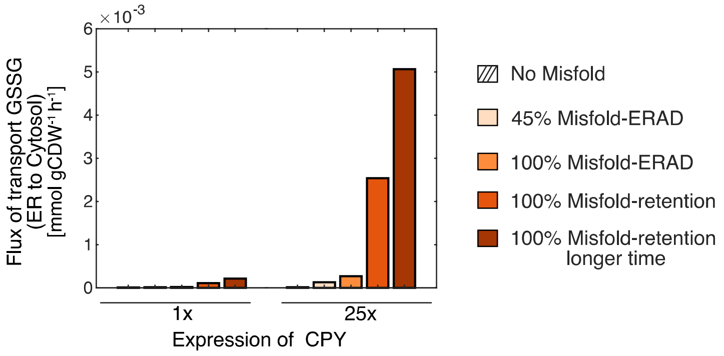

Supplementary Figure 4. Simulated fluxes for transporting Glutathione disulfide (GSSG) from ER to cytosol in expression of different amount CPY with different routes. 1x means its native expression level, 25x means 25-fold of its native expression level. Native expression level of CPY is from PaxDb database. Source data are provided as a Source Data file.

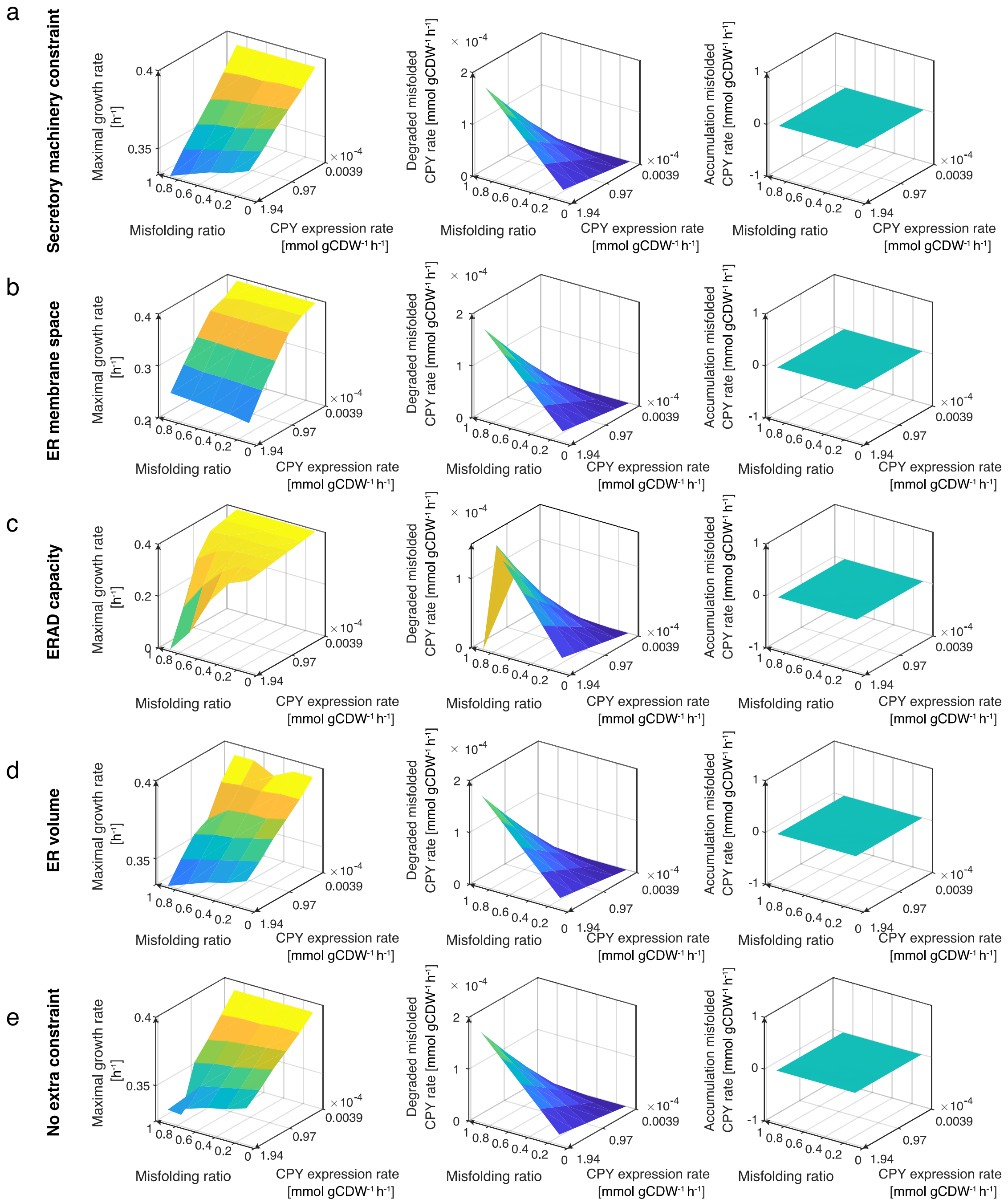

Supplementary Figure 5. Parameter analysis for the accumulation of misfolded CPY. Simulation with a) secretory machinery extra constraint, b) ER volume space extra constraint, c) ERAD capacity extra constraint and d) ER volume extra constraint. e) Simulation with no extra constraint. ER: Endoplasmic reticulum, ERAD: ER-associated degradation. Source data are provided as a Source Data file.

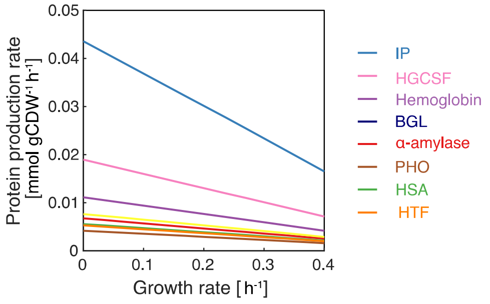

Supplementary Figure 6. Simulation of recombinant protein production under diverse specific growth rates using Yeast8 expanded with a reaction for production of the recombinant protein. Source data are provided as a Source Data file.

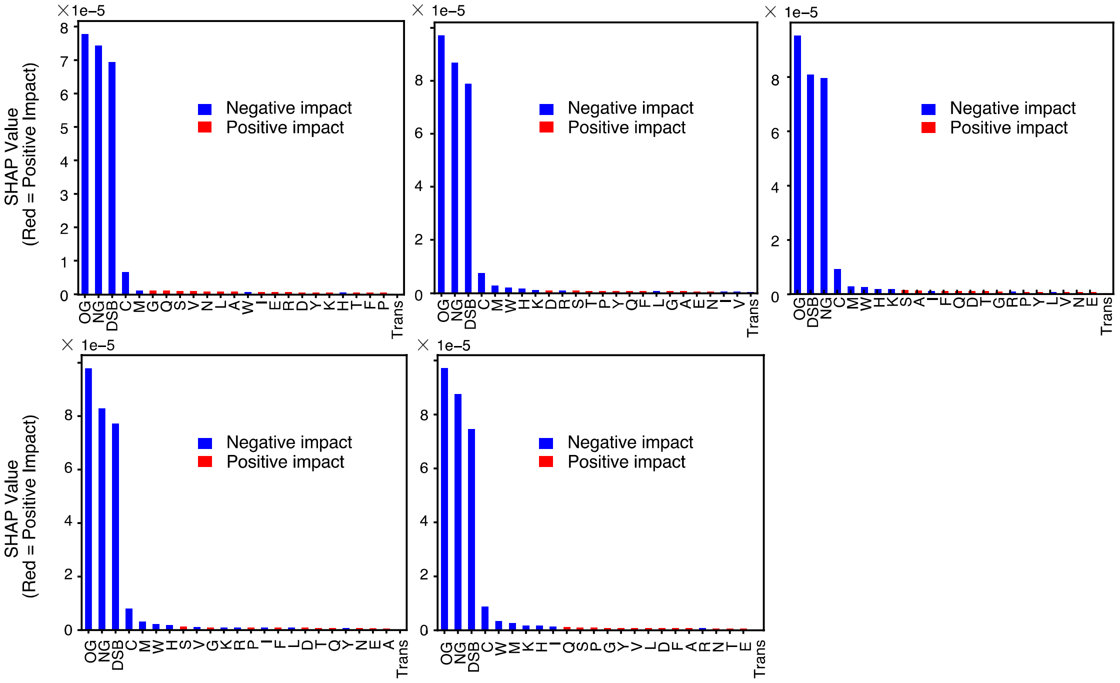

Supplementary Figure 7. Five-fold cross validation of the feature importance analysis. Source data are provided as a Source Data file.

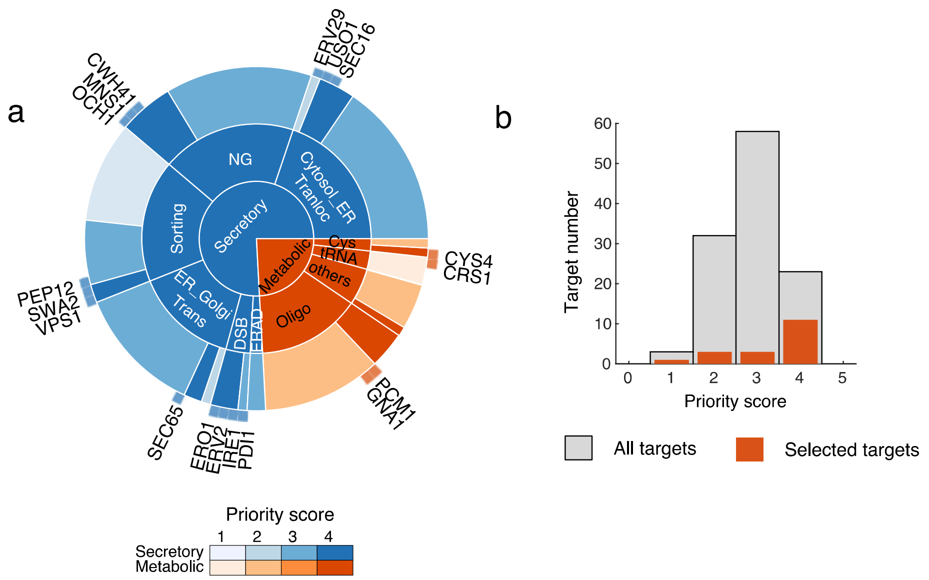

Supplementary Figure 8. Analysis of the overexpression targets for α-amylase overproduction. (a) Pathway analysis for predicted targets. Cys: Cysteine synthesis, tRNA: tRNA ligase, Oligo: oligosaccharide biosynthesis, ER_Golgi trans: ER to Golgi transport, Cytosol_ER transloc: Cytosol to ER translocation, NG: *N*-glycosylation, DSB: disulfide bond related pathway, ERAD: Endoplasmic reticulum (ER)-associated protein degradation. The outer circle represents the priority score for the target. Selected targets are denoted in the figure. (b) Priority score for predicted overexpression targets. Source data are provided as a Source Data file.

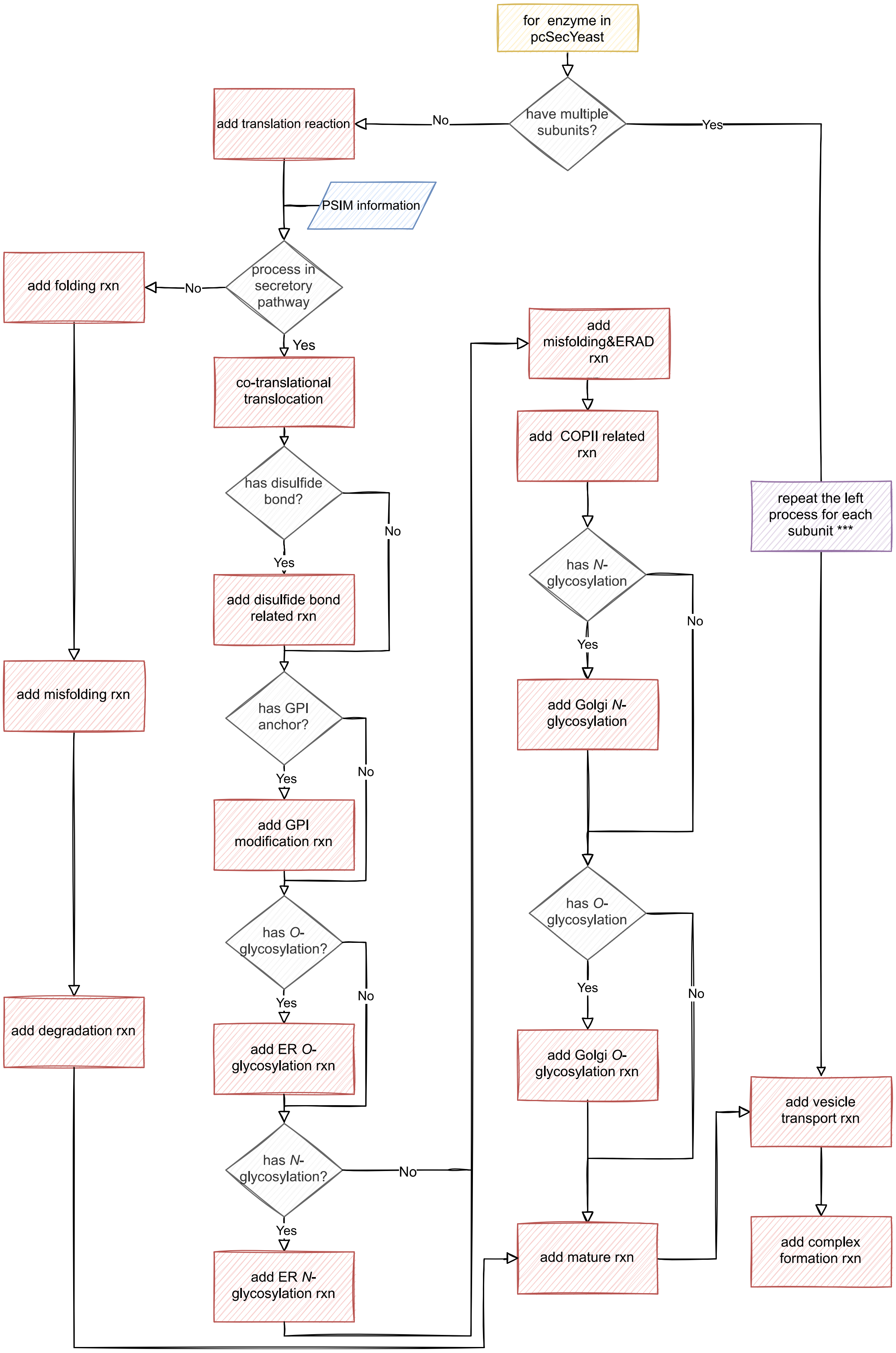

Supplementary Figure 9. Workflow for adding reactions for enzyme complexes in the pcSecYeast. *** repetitive process for all subunits in one enzyme complex.

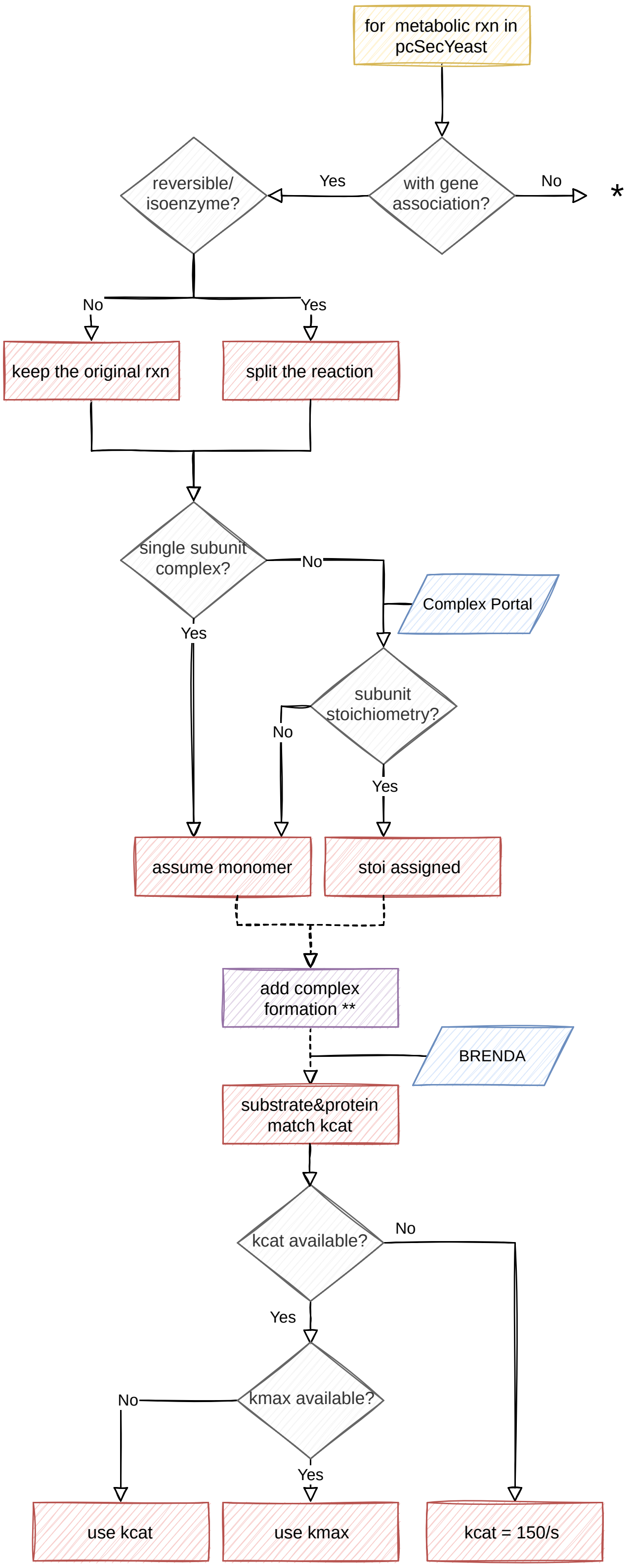

Supplementary Figure 10. Process of k_{cat} match for metabolic complexes. * For reactions does not have enzyme associated, we keep the original reaction in the model and do not add kinetic constraints for those reactions. ** Complex formation related process can be found in Supplementary Figure 9.

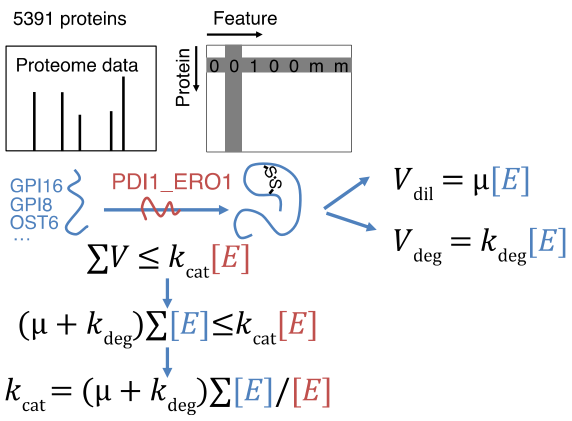

Supplementary Figure 11. Calculation of secretory machinery k_{cat} parameters.

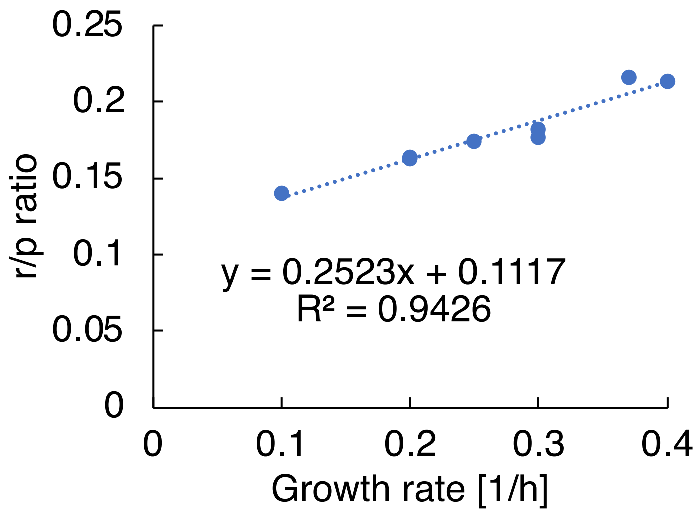

Supplementary Figure 12. Ribosome/protein ratio correlates with growth rates. Source data are provided as a Source Data file.

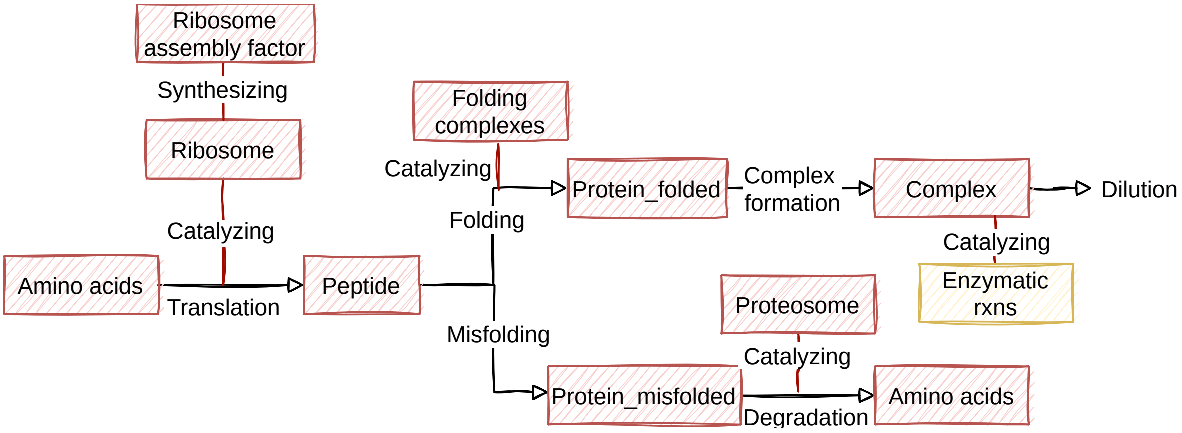

Supplementary Figure 13. Flowchart of the protein related process in the pcSecYeast.

Supplementary Table 1. Amino acid composition for α-amylase and the average *S. cerevisiae* protein.

Amino acid	mmol per g_α-amylase	mmol per g_cell protein*	Fold
Alanine	0.607	1.146	0.530
Arginine	0.18	0.401	0.449
Asparagine	0.426	0.254	1.677
Aspartic acid	0.689	0.743	0.927
Cysteine	0.148	0.016	8.980
Glutamine	0.279	0.263	1.060
Glutamic acid	0.197	0.754	0.261
Glycine	0.689	0.725	0.950
Histidine	0.115	0.166	0.695
Isoleucine	0.459	0.481	0.954
Leucine	0.558	0.740	0.754
Lysine	0.328	0.715	0.459
Methionine	0.148	0.127	1.169
Phenylalanine	0.213	0.334	0.637
Proline	0.344	0.411	0.836
Serine	0.59	0.463	1.274
Threonine	0.672	0.478	1.406
Tryptophan	0.164	0.071	2.313
Tyrosine	0.558	0.255	2.191
Valine	0.476	0.661	0.720

* is calculated from the protein pseudoreaction in the model.

Supplementary Table 2. Strains and plasmids used in this study.

Strains and plasmids	Genotype	Reference
Strains		
CEN.PK 530-1D	*MATa, ura3-52, HIS3, LEU2, TRP1, SUC2, MAL2-8^{c}, tpi1(41-707)::loxP-KanMX4-loxP*	PMID: 22265825
1D-AAC	CEN.PK 530-1D + pAlphaAmyCPOT	PMID: 24973076
Control	1D-AAC + pSPGM1	This study
SEC65	1D-AAC + pSPGM1-SEC65	This study
SWA2	1D-AAC + pSPGM1-SWA2	This study
MNS1	1D-AAC + pSPGM1-MNS1	This study
ERO1	1D-AAC + pSPGM1-ERO1	This study
IRE1	1D-AAC + pSPGM1-IRE1	This study
ERV2	1D-AAC + pSPGM1-ERV2	This study
CYS4	1D-AAC + pSPGM1-CYS4	This study
CRS1	1D-AAC + pSPGM1-CRS1	This study
PCM1	1D-AAC + pSPGM1-PCM1	This study
OCH1	1D-AAC + pSPGM1-OCH1	This study
GNA1	1D-AAC + pSPGM1-GNA1	This study
VPS1	1D-AAC + pSPGM1-VPS1	This study
PEP12	1D-AAC + pSPGM1-PEP12	This study
USO1	1D-AAC + pSPGM1-USO1	This study
Plasmids		
pAlphaAmyCPOT	2 µm, AmpR, *TPI1p-alpha factor leader-amylase gene-TPI1t, POT1* gene from *S. pombe* as a selection marker	PMID: 22179756
pSPGM1	2 µm, AmpR, *URA3*, *TEF1p-ADH1t*, *PGK1p-CYC1t*	PMID: 22487308
pSPGM1-SEC65	2 µm, AmpR, *URA3*, *TEF1p-SEC65-ADH1t*, *PGK1p-CYC1t*	This study
pSPGM1-SWA2	2 µm, AmpR, *URA3*, *TEF1p-SWA2-ADH1t*, *PGK1p-CYC1t*	This study
pSPGM1-MNS1	2 µm, AmpR, *URA3*, *TEF1p-MNS1-ADH1t*, *PGK1p-CYC1t*	This study
pSPGM1-ERO1	2 µm, AmpR, *URA3*, *TEF1p-ERO1-ADH1t*, *PGK1p-CYC1t*	This study
pSPGM1-IRE1	2 µm, AmpR, *URA3*, *TEF1p-IRE1-ADH1t*, *PGK1p-CYC1t*	This study
pSPGM1-ERV2	2 µm, AmpR, *URA3*, *TEF1p-ERV2-ADH1t*, *PGK1p-CYC1t*	This study
pSPGM1-CYS4	2 µm, AmpR, *URA3*, *TEF1p-CYS4-ADH1t*, *PGK1p-CYC1t*	This study
pSPGM1-CRS1	2 µm, AmpR, *URA3*, *TEF1p-CRS1-ADH1t*, *PGK1p-CYC1t*	This study
pSPGM1-PCM1	2 µm, AmpR, *URA3*, *TEF1p-PCM1-ADH1t*, *PGK1p-CYC1t*	This study
pSPGM1-OCH1	2 µm, AmpR, *URA3*, *TEF1p-OCH1-ADH1t*, *PGK1p-CYC1t*	This study
pSPGM1-GNA1	2 µm, AmpR, *URA3*, *TEF1p-GNA1-ADH1t*, *PGK1p-CYC1t*	This study
pSPGM1-VPS1	2 µm, AmpR, *URA3*, *TEF1p-VPS1-ADH1t*, *PGK1p-CYC1t*	This study
pSPGM1-PEP12	2 µm, AmpR, *URA3*, *TEF1p-PEP12-ADH1t*, *PGK1p-CYC1t*	This study
pSPGM1-USO1	2 µm, AmpR, *URA3*, *TEF1p-USO1-ADH1t*, *PGK1p-CYC1t*	This study

Supplementary Method 1. Model scope of pcSecYeast

pcSecYeast adopts the similar fine-grained proteome constrained concept as in the proteome constrained model pcYeast[1] and ME model yETFL[2], which is to couple the protein synthesis with the metabolic reactions through the enzyme kinetic capacity. The main difference of pcSecYeast with those models is that pcSecYeast has well-constructed processes for protein synthesis, folding, misfolding and degradation, which are rather lumped into one reaction or not covered in ME models or pc models. Therefore, pcSecYeast is more likely an expanded version of ME model/pc models in terms of the protein folding, misfolding, degradation process. Besides all applications of ME models or proteome constrained models, pcSecYeast allows the precise analysis of cellular behaviors for different physiological conditions, especially for the simulation of recombinant protein production.

The main difference of pcSecYeast with the whole cell model of yeast (WM_S288C[3]) is the process coverage. WM_S288C decomposes cell functionality into 26 cellular processes, while pcSecYeast covers six of those processes including metabolism, protein translation, protein folding, protein decay, protein modification and ribosome assembly. However, the protein folding and protein decay in pcSecYeast is more complex and protein-specific compared with that in WM-S288c and includes the secretion pathway in detail. As for the protein modification processes, glycosylation, disulfide bond and GPI considered in pcSecYeast are not considered in WM-S288C. Besides that, proteome-constrained models (including pcYeast[1] and yETFL[2] and pcSecYeast) are constraint-based optimization frameworks with the steady state assumption, while the whole cell model is a dynamic model which uses ordinary differential equations. Compared with the vast environment and condition dependent parameters requirement as in the WM-S288C[3], pcSecYeast requires less parameters, which enables a more efficient simulation of cell behavior.

Compared with other secretory models[4,5] which adopts basic GEM concept, pcSecYeast adopts the proteome constrained approach, which links the protein synthesis with the metabolism. This kind of expansion of protein synthesis can improve model prediction in terms of reduce variability of simulated fluxes, improved capability of complex phenotype prediction. Besides that, this kind of model can serve as the platform to integrate the transcriptome and proteome data for accurate flux simulation. PcSecYeast also covers more

processes compared with previous published secretory model, which is the current most comprehensive secretory model.

Supplementary Method 2. Information collection

Protein sequence

We downloaded protein sequence information from the UniProt database[6]. This information is stored as `Protein_sequence.mat` in the GitHub repository.

Protein information

All protein information was collected from the literature[7] and UniProt database[6]. For proteins that are annotated with multiple compartments, the first annotated compartment was used as its localization. For proteins that were denoted with signal peptide and the signal peptide length were not mentioned, we used the first 21 aa as its signal peptide.

Protein stoichiometry

For each functional protein, we should determine whether its functional unit is a monomer or oligomer. To do so, we collected protein stoichiometry information from the PDBe database (https://www.ebi.ac.uk/pdbe/) as well as the Complex Portal website (https://www.ebi.ac.uk/complexportal/home). This information is stored as `Protein_stoichiometry.mat` in the GitHub repository.

Supplementary Method 3. Reformulation of metabolic model

Metabolic model origin

The latest GEM Yeast8[8] for *S. cerevisiae* was used as the basis for the model pcSecYeast. Yeast8.3.5 was downloaded from the GitHub repository: https://github.com/SysBioChalmers/yeast-GEM.

Adding reactions for production of post-translational modification precursors

Yeast8.3.5 was firstly curated by adding reactions to ensure the production of all precursors for the secretory pathway. 92 reactions were added into Yeast8.3.5, which contains glycan synthesis, GPI anchor synthesis and transport reactions to shuttle currency metabolites between cytosol and other compartments in the secretory pathway. As for the GPI anchor synthesis, we chose *1-phosphatidyl-1D-myo-inositol* (1-16:0, 2-18:1) as the starting phosphatidyl-inositol according to the literature report[9]. Other lipids attached to GPI anchor were added according to the reference[10]. Those reactions can be found in the `Yeast8_Modification.xlsx` in the GitHub repository. Function `modifyYeast8` was used to add these reactions into Yeast8.

Splitting isoenzymes and reversible reactions

The updated model was then reformulated by splitting reversible reactions into forward and reverse reactions. Besides that, reactions catalyzed by isozymes were also split into multiple identical reactions with various isozymes. This step was performed to facilitate later k_{cat} match and enzyme constraining step. Function `splitModel` was used to perform this change.

Supplementary Method 4. Protein related process reconstruction

Peptide translation

We formulated the translation process for all proteins in the model. The substrates of translation include charged amino acid tRNAs, while the products include uncharged tRNA and the translated peptide. Besides the charged tRNAs, the translation process requires energy. Three steps in protein translation were considered: translation initiation, translation elongation, and translation termination. The translation initiation process requires one ATP molecule for binding mRNA with initiation factors, one ATP molecule for every step in the scanning process, and two GTP molecules for initiation factors[11]. The translation elongation process has been studied intensely in yeast[11]. In summary, for each amino acid, two ATPs and two GTPs are required during the elongation. As for the energy cost for the translation termination, one ATP and one GTP are required for each peptide. Translation initiation, elongation, and termination are lumped into one reaction in the model to improve simulation efficiency. The energy cost is calculated as 2N+3 ATP and 2N+3 GTP for each peptide, where N is the number of amino acids. The energy molecules were included as substrates in the translation reactions. Note that we simplified the model by assuming that all proteins are translated in the cytoplasm. Since the charged and uncharged tRNA are already metabolites in the original Yeast8, we did not update the tRNA charging process. The protein translation is catalyzed by the ribosome. We compiled all ribosome subunits from the reference[12]. The ribosome is assembled by ribosome assembly factors[12], which are also complied as a complex in the model.

Example of translation reaction for protein YAL012W:

```
reaction id: r_YAL012W_peptide_translation
reaction equation: 1582 H2O[c] + 791 ATP[c] + 791 GTP[c] + 41 Ala-tRNA(Ala)[c] + 13 Arg-tRNA(Arg)[c] + 22 Asn-tRNA(Asn)[c] + 21 Asp-tRNA(Asp)[c] + 15 Gln-tRNA(Gln)[c] + 21 Glu-tRNA(Glu)[c] + 28 Gly-tRNA(Gly)[c] + 14 His-tRNA(His)[c] + 24 Ile-tRNA(Ile)[c] + 40 Leu-tRNA(Leu)[c] + 22 Lys-tRNA(Lys)[c] + 4 Met-tRNA(Met)[c] + 14 Phe-tRNA(Phe)[c] + 17 Pro-tRNA(Pro)[c] + 29 Ser-tRNA(Ser)[c] + 28 Thr-tRNA(Thr)[c] + 2 Trp-tRNA(Trp)[c] + 10 Tyr-tRNA(Tyr)[c] + 30 Val-tRNA(Val)[c] -> 1582 H+[c] + 1582 phosphate[c] + 791 ADP[c] + 791 GDP[c] + 41 tRNA(Ala)[c] + 13 tRNA(Arg)[c] + 22 tRNA(Asn)[c] + 21 tRNA(Asp)[c] + 15 tRNA(Gln)[c] + 21 tRNA(Glu)[c] + 28 tRNA(Gly)[c] + 14 tRNA(His)[c] + 24 tRNA(Ile)[c] + 40 tRNA(Leu)[c] + 22 tRNA(Lys)[c] + 4 tRNA(Met)[c] + 14 tRNA(Phe)[c] + 17 tRNA(Pro)[c] + 29 tRNA(Ser)[c] + 28 tRNA(Thr)[c] + 2 tRNA(Trp)[c] + 10 tRNA(Tyr)[c] + 30 tRNA(Val)[c] + YAL012W_peptide[c]
catalyst: Mach_Ribosome_complex
```

Synthesis of ribosome:

reaction id: Mach_Ribosome_complex_formation

reaction equation: YGR214W_folding[c] + YLR048W_folding[c] + YLR441C_folding[c] + YML063W_folding[c] + YGL123W_folding[c] + YNL178W_folding[c] + YJR145C_folding[c] + YHR203C_folding[c] + YJR123W_folding[c] + YPL090C_folding[c] + YBR181C_folding[c] + YOR096W_folding[c] + YNL096C_folding[c] + YBL072C_folding[c] + YER102W_folding[c] + YPL081W_folding[c] + YBR189W_folding[c] + YOR293W_folding[c] + YMR230W_folding[c] + YDR025W_folding[c] + YBR048W_folding[c] + YOR369C_folding[c] + YDR064W_folding[c] + YCR031C_folding[c] + YJL191W_folding[c] + YOL040C_folding[c] + YMR143W_folding[c] + YDL083C_folding[c] + YML024W_folding[c] + YDR447C_folding[c] + YDR450W_folding[c] + YML026C_folding[c] + YOL121C_folding[c] + YNL302C_folding[c] + YHL015W_folding[c] + YKR057W_folding[c] + YJL136C_folding[c] + YJL190C_folding[c] + YLR367W_folding[c] + YGR118W_folding[c] + YPR132W_folding[c] + YER074W_folding[c] + YIL069C_folding[c] + YGR027C_folding[c] + YLR333C_folding[c] + YGL189C_folding[c] + YER131W_folding[c] + YKL156W_folding[c] + YHR021C_folding[c] + YLR264W_folding[c] + YLR388W_folding[c] + YDL061C_folding[c] + YLR287C_A_folding[c] + YOR182C_folding[c] + YLR167W_folding[n] + YMR116C_folding[c] + YPL220W_folding[c] + YGL135W_folding[c] + YFR031C_A_folding[c] + YIL018W_folding[c] + YOR063W_folding[c] + YBR031W_folding[c] + YDR012W_folding[c] + YPL131W_folding[c] + YML073C_folding[c] + YLR448W_folding[c] + YGL076C_folding[c] + YPL198W_folding[c] + YHL033C_folding[c] + YLL045C_folding[c] + YGL147C_folding[c] + YLR075W_folding[c] + YPR102C_folding[c] + YGR085C_folding[c] + YEL054C_folding[c] + YDR418W_folding[c] + YDL082W_folding[c] + YMR142C_folding[c] + YKL006W_folding[c] + YHL001W_folding[c] + YLR029C_folding[c] + YMR121C_folding[c] + YIL133C_folding[c] + YNL069C_folding[c] + YKL180W_folding[c] + YJL177W_folding[c] + YOL120C_folding[c] + YNL301C_folding[c] + YBR084C_A_folding[c] + YBL027W_folding[c] + YBR191W_folding[c] + YPL079W_folding[c] + YLR061W_folding[c] + YFL034C_A_folding[c] + YBL087C_folding[c] + YER117W_folding[c] + YGL031C_folding[c] + YGR148C_folding[c] + YOL127W_folding[c] + YLR344W_folding[c] + YGR034W_folding[c] + YHR010W_folding[c] + YDR471W_folding[c] + YGL103W_folding[c] + YFR032C_A_folding[c] + YGL030W_folding[c] + YDL075W_folding[c] + YLR406C_folding[c] + YBL092W_folding[c] + YPL143W_folding[c] + YOR234C_folding[c] + YER056C_A_folding[c] + YIL052C_folding[c] + YDL191W_folding[c] + YDL136W_folding[c] + YMR194W_folding[c] + YPL249C_A_folding[c] + YLR185W_folding[c] + YDR500C_folding[c] + YLR325C_folding[c] + YJL189W_folding[c] + YIL148W_folding[n] + YKR094C_folding[n] + YDL184C_folding[c] + YDL133C_A_folding[c] + YNL162W_folding[c] + YHR141C_folding[c] + YPR043W_folding[c] + YJR094W_A_folding[c] + YLR340W_folding[c] + YDL081C_folding[c] + YOL039W_folding[c] + YDR382W_folding[c] + YOR267C_folding[c] + YNL067W_folding[c] + YMR242C_folding[c] + YOR312C_folding[c] + YDL130W_folding[c] -> Mach_Ribosome_complex[c]

catalyst: Mach_Ribosome_Assembly_Factors_complex

Complex formation of ribosome assembly factors:

reaction id: Mach_Ribosome_Assembly_Factors_complex_formation

reaction equation: YAL005C_folding[c] + YBR247C_folding[n] + YCL031C_folding[n] + YCL059C_folding[n] + YCR057C_folding[n] + YEL026W_folding[n] + YER082C_folding[n] + YER127W_folding[n] + YHR148W_folding[n] + YHR196W_folding[n] + YIL035C_folding[c] + YKL143W_folding[c] + YKR060W_folding[n] + YDL014W_folding[n] + YDL148C_folding[n] + YDL153C_folding[n] + YDL213C_folding[n] + YDR324C_folding[n] + YDR339C_folding[n] + YDR449C_folding[n] + YGL120C_folding[n] + YGR090W_folding[n] + YGR128C_folding[n] + YJL010C_folding[n] + YJL069C_folding[n] + YJL109C_folding[n] + YJR002W_folding[n] + YLL011W_folding[n] + YLR129W_folding[n] + YLR186W_folding[n] + YLR197W_folding[n] + YLR222C_folding[n] + YLR409C_folding[n] + YLR430W_folding[n] + YML093W_folding[n] + YMR093W_folding[n] + YMR128W_folding[n] + YMR229C_folding[n] + YMR290C_folding[n] + YNL075W_folding[n] + YOL010W_folding[n] + YOR004W_folding[m] + YOR078W_folding[n] + YOR310C_folding[n] + YPL126W_folding[n] + YPL217C_folding[c] + YPR137W_folding[n] + YPR144C_folding[n] + YAL025C_folding[n] + YBR267W_folding[c] + YCL054W_folding[n] + YCR072C_folding[n] + YER002W_folding[n] + YER006W_folding[n] + YER126C_folding[n] + YHR052W_folding[n] + YHR066W_folding[n] + YHR088W_folding[n] + YHR170W_folding[c] + YIR012W_folding[c] + YIR026C_folding[n] + YKL009W_folding[n] + YKL082C_folding[n] + YKL172W_folding[n] + YKL186C_folding[n] + YKR081C_folding[n] + YDR087C_folding[n] + YDR101C_folding[c] + YDR312W_folding[n] + YGL029W_folding[n] + YGL099W_folding[c] + YGL111W_folding[n] + YGL173C_folding[c] + YGR103W_folding[n] + YGR245C_folding[n] + YJL050W_folding[n] + YLR002C_folding[n] + YLR009W_folding[c] + YLR074C_folding[c] + YLR106C_folding[n] + YLR175W_folding[n] + YLR387C_folding[c] + YLR449W_folding[n] + YML074C_folding[n] + YMR049C_folding[n] + YNL002C_folding[n] + YNL061W_folding[n] + YNL110C_folding[c] + YNR053C_folding[n] + YOL077C_folding[n] + YOR272W_folding[n] + YOR294W_folding[n] + YPL093W_folding[n] + YPL146C_folding[n] + YPL169C_folding[c] + YPL211W_folding[c] + YPR016C_folding[c] + YPR143W_folding[n] + YBL004W_folding[c] + YEL055C_folding[n] + YHR065C_folding[n] + YHR169W_folding[n] + YIL091C_folding[n] + YKL078W_folding[n] + YKL099C_folding[n] + YDR021W_folding[n] + YDR365C_folding[n] + YGL019W_folding[c] + YGL171W_folding[n] + YGR081C_folding[n] + YGR159C_folding[n] + YGR251W_folding[n] + YGR280C_folding[n] + YJL033W_folding[n] + YLR068W_folding[n] + YLR336C_folding[n] + YNL224C_folding[c] + YNR054C_folding[n] + YOR039W_folding[c] + YOR061W_folding[c] + YOR287C_folding[n] + YPR112C_folding[n] + YBR142W_folding[n] + YHR085W_folding[n] + YHR197W_folding[n] + YKL014C_folding[n] + YKL021C_folding[n] + YKR024C_folding[n] + YFL002C_folding[n] + YFR001W_folding[n] + YDL031W_folding[n] + YDR060W_folding[n] + YDR412W_folding[n] + YDR496C_folding[n] + YGL078C_folding[n] + YGL246C_folding[n] + YGR276C_folding[n] + YJL122W_folding[c] + YJR041C_folding[n] + YLL008W_folding[n] + YLL024C_folding[e] + YLL034C_folding[n] + YLR022C_folding[c] + YLR059C_folding[m] + YLR221C_folding[n]

+ YLR276C_folding[n] + YLR397C_folding[c] + YMR285C_folding[c] + YNL112W_folding[c] + YNL163C_folding[c] + YNL182C_folding[n] + YNL227C_folding[n] + YNR038W_folding[n] + YOL041C_folding[n] + YOL144W_folding[n] + YOR048C_folding[n] + YOR206W_folding[n] + YPL012W_folding[c] + YPL043W_folding[n] -> Mach_Ribosome_Assembly_Factors_complex[c]
catalyst: -

Function `addTranslationRxns` is used to add translation reactions for proteins.

Protein translocation

As for proteins that are processed by the secretory pathway, folding and further processing are described in detail as follows. The nascent translated peptide is then translocated into the ER for further modification. There are three different pathways for protein translocation from the cytosol to the ER: co-translational translocation, post-translational translocation, and post-translational translocation-tail- targeting[13,14].

Co-translational translocation in *S. cerevisiae* involves the interplay of the nascent peptide, the ribosome, the signal recognition particle (SRP), the signal recognition receptor (SR), and either the Ssh1 or the Sec61 translocon pore. SRP is a complex of six proteins (Srp14, Srp21, Srp54, Srp68, Srp72, and Sec65) and a 7S single RNA (SCR1)[15–17]. SRP interacts with the signal peptide of nascent peptides to form ribosome–nascent chain (RNC) complex, thus by the interaction of SPR and a signal receptor complex (SR), encoded by *SRP101* and *SRP102,* the RNC complex can attach to ER membrane. Finally, the RNC complex is transferred to the translocon, and then SRP and SR dissociate. Nascent peptides are then translocated into the ER. GTP bind to both SRP (via the Srp54 subunit) and the SR, which is critical for their interaction.

In the model, this process was divided into six template reactions. Only proteins with signal peptide were assumed to adopt this pathway in the model. If the signal peptide sequence has not been annotated in UniProt, we use the first 21 amino acids of the protein as the signal peptide. The degradation of signal peptide is described in further section together with protein degradation.

1. signal peptide recognition
2. ER receptor biding to peptide-SRPC
3. binding of peptide -SRPC-SRC to the translocator (Sec61C)
4. binding of peptide -SRPC-SRC to the translocator (Ssh1C)
5. signal peptidase
6. export the signal peptide out of ER for degradation

Example of co-translation reaction of YDR367W:

```
reaction id: YDR367W_co_translation_TC_sec_SRPC_complex
reaction equation: YDR367W_peptide[c] -> YDR367W_translocate_1[c]
catalyst: sec_SRPC_complex

reaction id: YDR367W_co_translation_TC_sec_SRC_complex
reaction equation: YDR367W_translocate_1[c] -> YDR367W_translocate_2[c]
catalyst: sec_SRC_complex

reaction id: YDR367W_co_translation_TC_sec_SEC61C_complex
reaction equation: 2 H2O[c] + 2 GTP[c] + YDR367W_translocate_2[c] -> H+[c] + 2 phosphate[c] + 2 GDP[c] +
YDR367W_translocate_3[c]
catalyst: sec_SEC61C_complex

reaction id: YDR367W_co_translation_TC_sec_SSH1C_complex
reaction equation: 2 H2O[c] + 2 GTP[c] + YDR367W_translocate_2[c] -> H+[c] + 2 phosphate[c] + 2 GDP[c] +
YDR367W_translocate_3[c]
catalyst: sec_SSH1C_complex

reaction id: YDR367W_co_translation_TC_sec_SPC_complex
reaction equation: H2O[c] + YDR367W_translocate_3[c] -> YDR367W[er] + YDR367W_sp[er]
catalyst: sec_SPC_complex

reaction id: YDR367W_export_sp_to_c
reaction equation: YDR367W_sp[er] -> YDR367W_sp[c]
catalyst: -
```

Enzyme complex formation for enzymes used in the co-translational translocation process:

reaction id: sec_SRPC_complex_formation
reaction equation: YDL092W_folding[n] + YKL122C_folding[n] + YPR088C_folding[c] + YML105C_folding[c] + YPL243W_folding[n] + YPL210C_folding[er] -> sec_SRPC_complex[erm]
catalyst: -

reaction id: sec_SRC_complex_formation
reaction equation: YDR292C_folding[erm] + YKL154W_folding[erm] -> sec_SRC_complex[erm]
catalyst: -

reaction id: sec_SEC61C_complex_formation
reaction equation: YER087C_B_folding[erm] + YLR378C_folding[erm] + YDR086C_folding[erm] -> sec_SEC61C_complex[erm]
catalyst: -

reaction id: sec_SSH1C_complex_formation
reaction equation: YDR086C_folding[erm] + YER019C_A_folding[erm] + YBR283C_folding[erm] -> sec_SSH1C_complex[erm]
catalyst: -

reaction id: sec_SPC_complex_formation
reaction equation: YIR022W_folding[erm] + YJR010C_A_folding[erm] + YML055W_folding[erm] + YLR066W_folding[erm] -> sec_SPC_complex[erm]
catalyst: -

Post-translational translocation is equivalently important with co-translational translocation. Nascent peptides exit the ribosome with the help of RAC chaperones (Ssb1, Ssz1 and Zuo1)[18]. After that, nascent peptides remain in an unfolded or loosely folded state, bind to the cytosolic chaperones Ssa1 and Ydj1 to avoid aggregation, which are then released before the translocation initiate. The translocation is mediated by the SEC complex, which comprises Sec61, Sbh1, Sss1, Sec62, Sec63, Sec71, and Sec72. The chaperon Kar2 in the ER lumen was suggested to drive the nascent protein into the ER[19].

In the model, this process was divided into four template reactions. The coefficient of ATP in step 4 was set as length/40, since the ATP molecule bound to the chaperone Kar2, is assumed to be hydrolyzed to ADP for every 40 amino acids that pass through the translocon pore[20].

1. exit the ribosome
2. bind to the cytosolic chaperone
3. translocation
4. pulling of nascent protein

Example of post-translation reaction of YDR453C:

```
reaction id: YDR453C_Post_translation_PSTA_sec_RAC_complex
reaction equation: YDR453C_peptide[c] -> YDR453C_translocate_1[c]
catalyst: sec_RAC_complex

reaction id: YDR453C_Post_translation_PSTA_sec_Ssa1_Ydj1_Snl1_complex
reaction equation: H2O[c] + ATP[c] + YDR453C_translocate_1[c] -> H+[c] + phosphate[c] + ADP[c] + YDR453C_translocate_2[c]
catalyst: sec_Ssa1_Ydj1_Snl1_complex

reaction id: YDR453C_Post_translation_PSTA_sec_SEC61SEC63C_complex
reaction equation: YDR453C_translocate_2[c] -> YDR453C_translocate_3[c]
catalyst: sec_SEC61SEC63C_complex

reaction id: YDR453C_Post_translation_PSTA_sec_BIP_NEFS_complex
reaction equation: 5 H2O[c] + 5 ATP[c] + YDR453C_translocate_3[c] -> 5 H+[c] + 5 phosphate[c] + 5 ADP[c] + YDR453C[er]
catalyst: sec_BIP_NEFS_complex
```

Enzyme complex formation for enzymes used in the post-translational translocation process:

```
reaction id: sec_RAC_complex_formation
reaction equation: YDL229W_folding[c] + YGR285C_folding[c] + YHR064C_folding[c] -> sec_RAC_complex[c]
catalyst: -

reaction id: sec_Ssa1_Ydj1_Snl1_complex_formation
reaction equation: YAL005C_folding[c] + YNL064C_folding[c] + YIL016W_folding[erm] -> sec_Ssa1_Ydj1_Snl1_complex[erm]
catalyst: -
```

reaction id: sec_SEC61SEC63C_complex_formation

reaction equation: YER087C_B_folding[erm] + YLR378C_folding[erm] + YDR086C_folding[erm] + YBR171W_folding[erm] + YOR254C_folding[erm] + YLR292C_folding[c] + YPL094C_folding[erm] -> sec_SEC61SEC63C_complex[erm]

catalyst: -

reaction id: sec_BIP_NEFS_complex_formation

reaction equation: YJL034W_folding[er] + YOL031C_folding[er] + YKL073W_folding[er] -> sec_BIP_NEFS_complex[er]

catalyst: -

Post-translational translocation-tail targeting is a unique translocation process, especially for tail-anchored (TA) proteins[14]. This process is also termed as the GET pathway, which involves Sgt2, Get4, Get5 and Get3 proteins. This process initiates from loading the TA proteins from the ribosome by the complex composed of Sgt2, Get4 and Get5. Then, the complex binds to Get3, a cytosolic transmembrane domains (TMD) recognition complex. This Get3 complex delivers the protein to the ER receptor composed of Get1 and Get2.

This process was formulated into three template reactions in the model, proteins with GPI anchor adopt this pathway for translocation.

1. load the TA proteins
2. bind to Get3
3. bind to ER receptor

Example of post-translation reaction-tail-targeting of YGR216C:

reaction id: YGR216C_Post_translation_PSTB_sec_Sgt2_Get4_Get5_complex

reaction equation: YGR216C_peptide[c] -> YGR216C_translocate_1[c]

catalyst: sec_Sgt2_Get4_Get5_complex

reaction id: YGR216C_Post_translation_PSTB_sec_Get3_complex

reaction equation: H2O[c] + ATP[c] + YGR216C_translocate_1[c] -> H+[c] + phosphate[c] + ADP[c] + YGR216C_translocate_2[c]

catalyst: sec_Get3_complex

reaction id: YGR216C_Post_translation_PSTB_sec_Get1_Get2_complex

reaction equation: YGR216C_translocate_2[c] -> YGR216C[er]

catalyst: sec_Get1_Get2_complex

Complex formation for enzymes in post-translation reaction-tail-targeting pathway:

reaction id: sec_Sgt2_Get4_Get5_complex_formation

reaction equation: YOR007C_folding[c] + YOR164C_folding[c] + YOL111C_folding[c] -> sec_Sgt2_Get4_Get5_complex[c]

catalyst: -

reaction id: sec_Get3_complex_formation

reaction equation: 2 YDL100C_folding[c] -> sec_Get3_complex[c]

catalyst: -

reaction id: sec_Get1_Get2_complex_formation

reaction equation: 2 YGL020C_folding[erm] + 2 YER083C_folding[erm] -> sec_Get1_Get2_complex[erm]

catalyst: -

Disulfide bond formation

This process is required for proteins annotated with disulfide bonds. The nascent peptide is captured by the chaperone Kar2 to mediate folding. Sulfhydryl groups are then oxidized by protein disulfide isomerases (Pdi1). Reoxidation of Pdi1 is mediated by ER oxidoreductin (Ero1), which in turn transfers electrons to O_2, thereby generating reactive oxygen species (ROS)[13].

This step in the model was divided into two template reactions.

1. bind to the chaperone
2. disulfide bond formation

Example of disulfide bond formation of YCL035C:

reaction id: YCL035C_DSB_sec_BIP_NEFS_complex

reaction equation: 2.775 H2O[er] + 2.775 ATP[er] + YCL035C[er] -> 2.775 H+[er] + 2.775 phosphate[er] + 2.775 ADP[er] + YCL035C_Kar2ATPcplx[er]

catalyst: sec_BIP_NEFS_complex

reaction id: YCL035C_DSB_PDI_II_sec_PDI1_ERV2_Ero1p_complex

reaction equation: PDI-ox[er] + YCL035C_Kar2ATPcplx[er] -> PDI[er] + YCL035C_DSB[er]

catalyst: sec_PDI1_ERV2_Ero1p_complex

Complex formation for enzymes used in dilsulfide bond related process:

reaction id: sec_BIP_NEFS_complex_formation

reaction equation: YJL034W_folding[er] + YOL031C_folding[er] + YKL073W_folding[er] -> sec_BIP_NEFS_complex[er]

catalyst: -

reaction id: sec_PDI1_ERV2_Ero1p_complex_formation

reaction equation: YCL043C_folding[er] + YML130C_folding[erm] + YPR037C_folding[erm] -> sec_PDI1_ERV2_Ero1p_complex[er]

catalyst: sec_PDI1_ERV2_Ero1p_complex

GPI formation

GPI formation process was formulated into several reactions according to reference[9]. Metabolic reactions for glycosylphosphatidylinositols (GPIs) synthesis were gap-filled in the Yeast8 modification step. Here, the first step is to transfer synthesized GPIs into proteins, followed by several remodelling of the sugar and lipid moieties in the GPI anchor. The acyl chain from the inositol is firstly removed by the Bst1, then the C18:1 fatty acid of the primary anchor is removed by a phospholipase A2 (Per1)[21]. Then, a C26:0 fatty acid is attached to the diacylglycerol moiety catalyzed by Gup1. For most GPI anchors, this modified diacylglycerol-based anchor is subsequently transformed into a ceramide-containing anchor catalyzed by Cwh43[22]. Ted1 further removes a phosphoethanolamine (PEtN) on the second mannose. Even though the GPI anchor is further modified in the Golgi, we do not include the Golgi modification part in the model as the enzyme responsible for this has not been identified. This could be easily added into the model in the future[23].

In the model, the GPI formation was divided into six steps:

1. GPI transfer
2. removal of the acyl chain from the inositol

3. removal of the unsaturated acyl chain at the sn-2 position of diacylglycerol to form lyso-GPI
4. transfer C26 saturated acyl chain to the sn-2 position
5. change the lipid moiety to ceramide consisting of PHS with a hydroxy-C26 fatty acid
6. removes a PEtN on the second mannose
7. GPI transfer with recycled GPI anchor from misfolded protein

Example of GPI formation and transfer of YDR437W:

reaction id: YDR437W_GPIRI_sec_GPIR_complex
reaction equation: 6-O-2-O-((2-aminoethyl)phosphoryl)-alpha-D-mannosyl-(1-2)-{alpha-D-mannosyl-2-O-((2-aminoethyl)phosphoryl)-(1-2)-alpha-D-mannosyl-(1-6)-2-O-((2-aminoethyl)phosphoryl)-alpha-D-mannosyl-(1-4)-alpha-D-glucosaminyl}-O-acyl-1-phosphatidyl-1D-myo-inositol[er] + YDR437W[er] -> H2O[er] + YDR437W_GPI_G1[er]
catalyst: sec_GPIR_complex

reaction id: YDR437W_GPIRII_sec_Bst1p_complex
reaction equation: YDR437W_GPI_G1[er] -> palmitate[erm] + YDR437W_GPI_G2[er]
catalyst: sec_Bst1p_complex

reaction id: YDR437W_GPIRIII_sec_Per1p_complex
reaction equation: H2O[er] + YDR437W_GPI_G2[er] -> oleate[er] + YDR437W_GPI_G3[er]
catalyst: sec_Per1p_complex

reaction id: YDR437W_GPIRIV_sec_Gup1p_complex
reaction equation: hexacosanoyl-CoA[er] + YDR437W_GPI_G3[er] -> H+[er] + coenzyme A[er] + YDR437W_GPI_G4[er] catalyst: sec_Gup1p_complex

reaction id: YDR437W_GPIRV_sec_Cwh43p_Gpi7p_Mcd4p_complex
reaction equation: ceramide-3 (C26)[er] + YDR437W_GPI_G4[er] -> diglyceride (1-26:0, 2-16:0)[er] + YDR437W_GPI_G5[er]
catalyst: sec_Cwh43p_Gpi7p_Mcd4p_complex

reaction id: YDR437W_GPIRVI_sec_Ted1p_complex
reaction equation: H2O[er] + YDR437W_GPI_G5[er] -> O-phosphoethanolamine[er] + YDR437W_GPI_G6[er]
catalyst: sec_Ted1p_complex

reaction id: YDR437W_GPIRIB_sec_GPIR_complex

reaction equation: 6-O-2-O-alpha-D-mannosyl-(1-2)-{alpha-D-mannosyl-2-O-((2-aminoethyl)phosphoryl)-(1-2)-alpha-D-mannosyl-(1-6)-2-O-((2-aminoethyl)phosphoryl)-alpha-D-mannosyl-(1-4)-alpha-D-glucosaminyl}-O-inositol-P-ceramide C (C26)[er] + YDR437W[er] -> H2O[er] + YDR437W_GPI_G6[er]

catalyst: sec_GPIR_complex

Complex formation of GPI related enzymes:

reaction id: sec_GPIR_complex_formation

reaction equation: 2 YLR088W_folding[erm] + 2 YDR331W_folding[erm] + 2 YHR188C_folding[erm] + 2 YDR434W_folding[erm] + 2 YLR459W_folding[erm] -> sec_GPIR_complex[erm]

catalyst: -

reaction id: sec_Bst1p_complex_formation

reaction equation: YFL025C_folding[erm] -> sec_Bst1p_complex[erm]

catalyst: -

reaction id: sec_Per1p_complex_formation

reaction equation: YCR044C_folding[erm] -> sec_Per1p_complex[erm]

catalyst: -

reaction id: sec_Gup1p_complex_formation

reaction equation: YGL084C_folding[erm] -> sec_Gup1p_complex[erm]

catalyst: -

reaction id: sec_Cwh43p_Gpi7p_Mcd4p_complex_formation

reaction equation: YKL165C_folding[erm] + YJL062W_folding[erm] + YCR017C_folding[erm] -> sec_Cwh43p_Gpi7p_Mcd4p_complex[erm]

catalyst: -

reaction id: sec_Ted1p_complex_formation

reaction equation: YIL039W_folding[erm] -> sec_Ted1p_complex[erm]

catalyst: -

reaction id: sec_GPIR_complex_formation

reaction equation: 2 YLR088W_folding[erm] + 2 YDR331W_folding[erm] + 2 YHR188C_folding[erm] + 2 YDR434W_folding[erm] + 2 YLR459W_folding[erm] -> sec_GPIR_complex[erm]

catalyst: -

ER *O*-glycosylation

In yeast, *O*-glycosylation initiates in the ER and extends in Golgi. This section describes how the ER *O*-glycosylation was formulated in the model. The glycan extension in Golgi will be described in the following sections when the peptide is transported into Golgi. More than six proteins in ER are responsible for protein manosylation in yeast. Those proteins make up the complex PMTC, protein O-mannosyltransferase, which transfers mannose residues from dolichyl phosphate D-mannose to protein Ser/Thr residues[24].

In the model, this process was formulated into one reaction:

1. ER *O*-glycosylation

Example of ER *O*-glycosylation of YJL137C:

reaction id: YJL137C_OG_EROG_sec_Pmt2p_Pmt5p_Pmt1p_Pmt6p_Pmt4p_Pmt3p_complex<br>reaction equation: 3 dolichyl D-mannosyl phosphate[er] + YJL137C[er] -> 3 dolichyl phosphate[er] + YJL137C_OG_M1[er]<br>catalyst: sec_Pmt2p_Pmt5p_Pmt1p_Pmt6p_Pmt4p_Pmt3p_complex

Complex formation of ER *O*-glycosylation related enzymes:

reaction id: sec_Pmt2p_Pmt5p_Pmt1p_Pmt6p_Pmt4p_Pmt3p_complex_formation<br>reaction equation: YAL023C_folding[erm] + YDL095W_folding[erm] + YDL093W_folding[erm] + YJR143C_folding[erm] + YOR321W_folding[erm] + YGR199W_folding[erm] -> sec_Pmt2p_Pmt5p_Pmt1p_Pmt6p_Pmt4p_Pmt3p_complex[erm]<br>catalyst: -

ER *N*-glycosylation

N-glycosylation is one of the most abundant post-translational modifications in yeast. The first step of *N*-glycosylation is to transfer the oligosaccharide precursor $Glc_3Man_9GlcNAc_2$ to the protein, mediated by the OSTC complex[25]. After that, three glucose residues in the glycan are trimmed by Cwh41[26] and Rot2[25]. Then one of the mannose residues added by Alg9 from *N*-linked core oligosaccharides is further trimmed by Mns1[25], which is the last trimming reaction that occurs in the ER before correctly-folded proteins migrate to the Golgi. The further glycan extension in Golgi will be described in the following part when the peptide is

transported into Golgi. The oligosaccharide precursor synthesis was added to the model in the previous Yeast8 modification step.

This process was divided into five steps in the model.

1. OSTC_complex ER *N*-glycan transfer
2. ER Glycan trimming I
3. ER Glycan trimming II
4. ER Glycan trimming III
5. ER demanosylation I

Example: ER *N*-glycosylation for YJL139C

reaction id: YJL139C_ERNG_NG_sec_OSTC_complex
reaction equation: 5 Glucose(3)Mannose(9)GlucoseNAc(2)-PP-dolichol[er] + YJL139C[er] -> 5 dolichyl phosphate[er] + YJL139C_G3M9[er]
catalyst: sec_OSTC_complex

reaction id: YJL139C_ERNG_FLI_NG_sec_Cwh41p_complex
reaction equation: 5 H2O[er] + YJL139C_G3M9[er] -> 5 D-glucose[er] + YJL139C_G2M9[er]
catalyst: sec_Cwh41p_complex

reaction id: YJL139C_ERNG_FLII_NG_sec_Rot2p_complex
reaction equation: 5 H2O[er] + YJL139C_G2M9[er] -> 5 D-glucose[er] + YJL139C_G1M9[er]
catalyst: sec_Rot2p_complex

reaction id: YJL139C_ERNG_FLIII_NG_sec_Rot2p_complex
reaction equation: 5 H2O[er] + YJL139C_G1M9[er] -> 5 D-glucose[er] + YJL139C_M9[er]
catalyst: sec_Rot2p_complex

reaction id: YJL139C_ERNG_FLIV_NG_sec_Mns1p_complex
reaction equation: 5 H2O[er] + YJL139C_M9[er] -> 5 D-mannose[er] + YJL139C_M8[er]
catalyst: sec_Mns1p_complex

Complex formation of enzymes used in ER *N*-glycosylation:

reaction id: sec_OSTC_complex_formation

reaction equation: YGL022W_folding[er] + YJL002C_folding[erm] + YEL002C_folding[erm] + YOR085W_folding[erm] + YML019W_folding[erm] + YMR149W_folding[erm] + YOR103C_folding[er] + YGL226C_A_folding[er] + YDL232W_folding[erm] -> sec_OSTC_complex[erm]

catalyst: -

reaction id: sec_Cwh41p_complex_formation

reaction equation: YGL027C_folding[erm] -> sec_Cwh41p_complex[erm]

catalyst: -

reaction id: sec_Rot2p_complex_formation

reaction equation: YBR229C_folding[er] -> sec_Rot2p_complex[er]

catalyst: -

reaction id: sec_Mns1p_complex_formation

reaction equation: YJR131W_folding[erm] -> sec_Mns1p_complex[erm]

catalyst: -

Misfolding and ERAD

Protein misfolding is a common cellular process that can produce intrinsically harmful products. In order to reduce the risk, the cell develops a highly efficient system for protein quality control and endoplasmic reticulum-associated degradation (ERAD) for the degradation of misfolded proteins. The ubiquitin-proteasome system (UPS) is a pathway in the cell responsible for the degradation of proteins. The pathway can be split into two main processes: ubiquitination and degradation. The ubiquitination process requires three enzymes: E1, E2 and E3[27]. The activating enzyme E1 activates a ubiquitin with one ATP molecule. The activated ubiquitin conjugates enzyme E2. The ubiquitin transfers to the ligase E3 from E2 and then to the misfolded protein when E3 binds to the misfolded protein targeted for degradation. The process repeats itself until the misfolded protein acquires a chain of ubiquitin with at least four ubiquitin long. Then, the tagged misfolded protein can then be released from E3 and recognized by the proteasome. In yeast, there are mainly three ERAD pathways: ERAD-C, ERAD-L and ERAD-M, they differ in the E3 ligase part. Both ERAD-L and ERAD-M uses Hrd1 ubiquitin ligase complex (Hrd1, Hrd3 and Der1), but luminal factor Yos9 seems dispensable for ERAD-M. ERAD-C uses the Doa10 ubiquitin ligase complex[28,29]. It is also

suggested that Doa10 is also the ubiquitin ligase for ERAD-M[30]. Thus, as for ERAD-M pathway, we added alternative pathways which can utilize either the Hrd1 or the Doa10 as the ubiquitin ligase in the model. All protein modifications such as disulfide bond, *N*-glycosylation and *O*-glycosylation are reversed in ERAD pathways, which are formulated as the first step in the ERAD pathways in the model. In order to represent the accumulation of misfolded protein, we added two extra reactions to reflect the occupation of misfolded protein with Kar2 and Pdi1[31].

The misfolding and ERAD is divided into reactions:

1. add Kar2 to the misfolded proteins
2. break the disulfide bond
3. trim one mannose off from the glycan
4. trim the GPI anchor
5. ERAD E3 ligase
6. ERAD ubiquitination
7. trim the glycan
8. misfolding protein degradation
9. misfolding protein accumulation with occupation of Pdi1
10. misfolding protein accumulation with occupation of Kar2

Example of misfolding and ERAD (since different proteins adopt different reactions based on their protein properties, therefore several proteins are used in this example):

reaction id: YJL139C_misfold_ERAD_sec_Kar2p_complex

reaction equation: 11 H2O[er] + 11 ATP[er] + YJL139C_M9[er] -> 11 H+[er] + 11 phosphate[er] + 11 ADP[er] + YJL139C_M9_misf[er]

catalyst: sec_Kar2p_complex

reaction id: YJR104C_ERAD2A_sec_Pdi1p_complex

reaction equation: 4 glutathione[er] + YJR104C_DSB_misf[er] -> 4 H+[er] + 2 glutathione disulfide[er] + YJR104C_DSB_misf_G1[er]

catalyst: sec_Pdi1p_complex

reaction id: YJL139C_ERAD2B

reaction equation: YJL139C_M9_misf[er] -> YJL139C_M9_misf_G1[er]

catalyst: -

reaction id: YJL139C_ERAD3A_sec_Mns1p_complex

reaction equation: 5 H2O[er] + YJL139C_M9_misf_G1[er] -> 5 D-mannose[er] + YJL139C_M9_misf_G2[er]

catalyst: sec_Mns1p_complex

reaction id: YJR104C_ERAD3B

reaction equation: YJR104C_DSB_misf_G1[er] -> YJR104C_DSB_misf_G2[er]

catalyst: -

reaction id: YJL139C_ERAD4A_sec_Mnl1p_Pdi1p_complex

reaction equation: 5 H2O[er] + YJL139C_M9_misf_G2[er] -> 5 D-mannose[er] + YJL139C_M9_misf_G3[er]

catalyst: sec_Mnl1p_Pdi1p_complex

reaction id: YJR104C_ERAD4B

reaction equation: YJR104C_DSB_misf_G2[er] -> YJR104C_DSB_misf_G3[er]

catalyst: -

reaction id: YKL165C_ERAD5A

reaction equation: H2O[er] + YKL165C_GPI_G6_M9_misf_G3[er] -> 6-O-2-O-alpha-D-mannosyl-(1-2)-{alpha-D-mannosyl-2-O-((2-aminoethyl)phosphoryl)-(1-2)-alpha-D-mannosyl-(1-6)-2-O-((2-aminoethyl)phosphoryl)-alpha-D-mannosyl-(1-4)-alpha-D-glucosaminyl}-O-inositol-P-ceramide C (C26)[er] + YKL165C_GPI_G6_M9_misf_G4[er]

catalyst: -

reaction id: YJL139C_ERAD5B

reaction equation: YJL139C_M9_misf_G3[er] -> YJL139C_M9_misf_G4[er]

catalyst: -

reaction id: YJL139C_ERADL_sec_Cue1p_Ubc6p_Ubc7p_Yos9p_Hrd1p_Hrd3p_Der1p_Usa1p_complex

reaction equation: YJL139C_M9_misf_G4[er] -> YJL139C_M9_misf_G5[er]

catalyst: sec_Cue1p_Ubc6p_Ubc7p_Yos9p_Hrd1p_Hrd3p_Der1p_Usa1p_complex

reaction id: YJL139C_ERADL_sec_Sbh1p_Sss1p_Ssh1p_Cdc48p_Ubx2p_Ufd1p_Npl4p_complex

reaction equation: 8 Ubiquitin_for_Transfer[c] + YJL139C_M9_misf_G5[er] -> 8 Ubiquitin[c] + YJL139C_M9_misf_G6[c]

catalyst: sec_Sbh1p_Sss1p_Ssh1p_Cdc48p_Ubx2p_Ufd1p_Npl4p_complex

reaction id: YKL165C_ERADM_sec_Cue1p_Ubc6p_Ubc7p_Hrd1p_Hrd3p_Der1p_complex

reaction equation: YKL165C_GPI_G6_M9_misf_G4[er] -> YKL165C_GPI_G6_M9_misf_G5[er]

catalyst: sec_Cue1p_Ubc6p_Ubc7p_Hrd1p_Hrd3p_Der1p_complex

reaction id: YKL165C_ERADM2_sec_Cue1p_Ubc6p_Ubc7p_Doa10p_complex

reaction equation: YKL165C_GPI_G6_M9_misf_G4[er] -> YKL165C_GPI_G6_M9_misf_G5[er]

catalyst: sec_Cue1p_Ubc6p_Ubc7p_Doa10p_complex

reaction id: YKL165C_ERADM_sec_Sbh1p_Sss1p_Ssh1p_Cdc48p_Ubx2p_Ufd1p_Npl4p_complex

reaction equation: 8 Ubiquitin_for_Transfer[c] + YKL165C_GPI_G6_M9_misf_G5[er] -> 8 Ubiquitin[c] + YKL165C_GPI_G6_M9_misf_G6[c]

catalyst: sec_Sbh1p_Sss1p_Ssh1p_Cdc48p_Ubx2p_Ufd1p_Npl4p_complex

reaction id: YNL038W_ERADC_sec_Cue1p_Ubc6p_Ubc7p_Doa10p_complex

reaction equation: YNL038W_GPI_G6_misf_G4[er] -> YNL038W_GPI_G6_misf_G5[er]

catalyst: sec_Cue1p_Ubc6p_Ubc7p_Doa10p_complex

reaction id: YNL038W_ERADC_sec_Sbh1p_Sss1p_Ssh1p_Cdc48p_Ubx2p_Ufd1p_Npl4p_complex

reaction equation: 8 Ubiquitin_for_Transfer[c] + YNL038W_GPI_G6_misf_G5[er] -> 8 Ubiquitin[c] + YNL038W_GPI_G6_misf_G6[c]

catalyst: sec_Sbh1p_Sss1p_Ssh1p_Cdc48p_Ubx2p_Ufd1p_Npl4p_complex

reaction id: YJL139C_ERAD7A_sec_Dsk2p_Rad23p_Png1p_Uba1p_complex

reaction equation: YJL139C_M9_misf_G6[c] -> 10 N-acetyl-alpha-D-glucosamine 1-phosphate[c] + 35 D-mannose[er] + YJL139C_misfolding[c]

catalyst: sec_Dsk2p_Rad23p_Png1p_Uba1p_complex

reaction id: YKR058W_ERAD7B_sec_Dsk2p_Rad23p_Png1p_Uba1p_complex

reaction equation: YKR058W_OG_M1_misf_G6[c] -> 2 D-mannose[er] + YKR058W_misfolding[c]

catalyst: sec_Dsk2p_Rad23p_Png1p_Uba1p_complex

reaction id: YNL038W_ERAD7C_sec_Dsk2p_Rad23p_Uba1p_complex

reaction equation: YNL038W_GPI_G6_misf_G6[c] -> YNL038W_misfolding[c]

catalyst: sec_Dsk2p_Rad23p_Uba1p_complex

reaction id: YJL139C_degradation_misfolding_c

reaction equation: YJL139C_misfolding[c] -> YJL139C_subunit[c]

catalyst: -

reaction id: YPL091W_cycle_accumulation_sec_pdi1p_ero1p_complex

reaction equation: 10 oxygen[er] + 20 glutathione[er] + YPL091W_DSB_misf[er] -> 10 glutathione disulfide[er] + 10 hydrogen peroxide[er] + YPL091W_DSB_misf2[er]

catalyst: sec_pdi1p_ero1p_complex

reaction id: YPL091W_cycle_accumulation_sec_acc_Kar2p_complex

reaction equation: 120 H2O[er] + 120 ATP[er] + YPL091W_DSB_misf2[er] -> 120 H+[er] + 120 phosphate[er] + 120 ADP[er] + YPL091W_DSB_misfolding_acc[er]

catalyst: sec_acc_Kar2p_complex

Complex formation for enzymes in ERAD pathways

reaction id: sec_Kar2p_complex_formation

reaction equation: YJL034W_folding[er] -> sec_Kar2p_complex[er]

catalyst: -

reaction id: sec_Pdi1p_complex_formation

reaction equation: 2 YCL043C_folding[er] -> sec_Pdi1p_complex[er]

catalyst: -

reaction id: sec_Mns1p_complex_formation

reaction equation: YJR131W_folding[erm] -> sec_Mns1p_complex[erm]

catalyst: -

reaction id: sec_Mnl1p_Pdi1p_complex_formation

reaction equation: 2 YCL043C_folding[er] + YHR204W_folding[er] -> sec_Mnl1p_Pdi1p_complex[er]

catalyst: -

reaction id: sec_Cue1p_Ubc6p_Ubc7p_Yos9p_Hrd1p_Hrd3p_Der1p_Usa1p_complex_formation

reaction equation: YMR264W_folding[erm] + YMR022W_folding[erm] + 2 YDR057W_folding[erm] + YLR207W_folding[erm] + 2 YOL013C_folding[erm] + YER100W_folding[erm] + YBR201W_folding[erm] + YML029W_folding[erm] -> sec_Cue1p_Ubc6p_Ubc7p_Yos9p_Hrd1p_Hrd3p_Der1p_Usa1p_complex[erm]

catalyst: -

reaction id: sec_Sbh1p_Sss1p_Ssh1p_Cdc48p_Ubx2p_Ufd1p_Npl4p_complex_formation

reaction equation: YER087C_B_folding[erm] + YDR086C_folding[erm] + YBR283C_folding[erm] + 6 YDL126C_folding[er] + YGR048W_folding[erm] + YBR170C_folding[er] + YML013W_folding[erm] -> sec_Sbh1p_Sss1p_Ssh1p_Cdc48p_Ubx2p_Ufd1p_Npl4p_complex[erm]

catalyst: -

reaction id: sec_Cue1p_Ubc6p_Ubc7p_Hrd1p_Hrd3p_Der1p_complex_formation

reaction equation: YMR264W_folding[erm] + YMR022W_folding[erm] + YLR207W_folding[erm] + 2 YOL013C_folding[erm] + YER100W_folding[erm] + YBR201W_folding[erm] -> sec_Cue1p_Ubc6p_Ubc7p_Hrd1p_Hrd3p_Der1p_complex[erm]

catalyst: -

reaction id: sec_Sbh1p_Sss1p_Ssh1p_Cdc48p_Ubx2p_Ufd1p_Npl4p_complex_formation

reaction equation: YER087C_B_folding[erm] + YDR086C_folding[erm] + YBR283C_folding[erm] + 6 YDL126C_folding[er] + YGR048W_folding[erm] + YBR170C_folding[er] + YML013W_folding[erm] -> sec_Sbh1p_Sss1p_Ssh1p_Cdc48p_Ubx2p_Ufd1p_Npl4p_complex[erm]

catalyst: -

reaction id: sec_Cue1p_Ubc6p_Ubc7p_Doa10p_complex_formation

reaction equation: YMR264W_folding[erm] + YMR022W_folding[erm] + YER100W_folding[erm] + YIL030C_folding[erm] -> sec_Cue1p_Ubc6p_Ubc7p_Doa10p_complex[erm]

catalyst: -

reaction id: sec_Sbh1p_Sss1p_Ssh1p_Cdc48p_Ubx2p_Ufd1p_Npl4p_complex_formation

reaction equation: YER087C_B_folding[erm] + YDR086C_folding[erm] + YBR283C_folding[erm] + 6 YDL126C_folding[er] + YGR048W_folding[erm] + YBR170C_folding[er] + YML013W_folding[erm] -> sec_Sbh1p_Sss1p_Ssh1p_Cdc48p_Ubx2p_Ufd1p_Npl4p_complex[erm]

catalyst: -

reaction id: sec_Dsk2p_Rad23p_Png1p_Uba1p_complex_formation

reaction equation: 4 YMR276W_folding[n] + YEL037C_folding[c] + YPL096W_folding[c] + 2 YKL210W_folding[c] -> sec_Dsk2p_Rad23p_Png1p_Uba1p_complex[c]

catalyst: -

reaction id: sec_Dsk2p_Rad23p_Png1p_Uba1p_complex_formation

reaction equation: 4 YMR276W_folding[n] + YEL037C_folding[c] + YPL096W_folding[c] + 2 YKL210W_folding[c] -> sec_Dsk2p_Rad23p_Png1p_Uba1p_complex[c]

reaction id: YNL038W_ERAD7C_sec_Dsk2p_Rad23p_Uba1p_complex

reaction equation: YNL038W_GPI_G6_misf_G4[er] -> YNL038W_GPI_G6_misf_G5[er]

catalyst: -

reaction id: sec_pdi1p_ero1p_complex_formation

reaction equation: 2 YCL043C_folding[er] + YML130C_folding[erm] -> sec_pdi1p_ero1p_complex[er]

catalyst: sec_pdi1p_ero1p_complex

reaction id: sec_acc_Kar2p_complex_formation

reaction equation: YJL034W_folding[er] -> sec_acc_Kar2p_complex[er]

catalyst: -

Protein and signal peptide degradation

Translocated misfolded proteins are then degraded into amino acids by the cytosol proteosome. Similar reactions are also added for the cleaved signal peptide. The energy cost for the degradation estimated for eukaryotes is around 0.25-1.3 ATP/aa (the calculation is based on an average protein length of 467 aa)[32]. We adopted the highest value of 1.3 ATP/aa as the energetic cost for protein degradation and signal peptide. To be noted here, even though that pre and pro sequence in the leader sequence of recombinant protein degraded in ER and Golgi, respectively[33]. To simplify this process, full leader sequence is cleaved in ER for degradation in the model.

Example of degradation of YPL091W:

reaction id: r_YPL091W_subunit_degradation

reaction equation: 627 H2O[c] + 627 ATP[c] + YPL091W_subunit[c] -> 627 H+[c] + 627 phosphate[c] + 35 L-glutamate[c] + 10 L-methionine[c] + 33 L-alanine[c] + 11 L-glutamine[c] + 627 ADP[c] + 24 L-aspartate[c] + 43 L-glycine[c] + 18 L-arginine[c] + 28 L-asparagine[c] + 29 L-serine[c] + 5 L-cysteine[c] + 15 L-histidine[c] + 32 L-isoleucine[c] + 14 L-proline[c] + 26 L-threonine[c] + 4 L-tryptophan[c] + 19 L-tyrosine[c] + 34 L-leucine[c] + 42 L-lysine[c] + 17 L-phenylalanine[c] + 45 L-valine[c]

catalyst: Mach_proteasome_complex

Example of degradation of signal peptide for YAL053W:

reaction id: r_YAL053W_SP_degradation

reaction equation: 28 H2O[c] + 28 ATP[c] + YAL053W_sp[c] -> 28 H+[c] + 28 phosphate[c] + L-methionine[c] + 2 L-alanine[c] + 28 ADP[c] + L-glycine[c] + L-arginine[c] + L-asparagine[c] + L-serine[c] + 3 L-cysteine[c] + L-isoleucine[c] + 3 L-threonine[c] + 4 L-leucine[c] + 3 L-phenylalanine[c] + L-valine[c]

catalyst: Mach_proteasome_complex

Complex formation of proteosome complex:

reaction id: Mach_proteasome_complex_formation

reaction equation: YMR276W_folding[n] + YEL037C_folding[c] + YJL001W_folding[c] + YOR157C_folding[c] + YER094C_folding[c] + YER012W_folding[c] + YPR103W_folding[c] + YBL041W_folding[c] + YFR050C_folding[c] + YGL011C_folding[c] + YML092C_folding[c] + YGR135W_folding[c] + YOL038W_folding[c] + YGR253C_folding[c] + YMR314W_folding[c] + YOR362C_folding[c] + YDL097C_folding[c] + YDL147W_folding[c] + YDR363W_A_folding[c] + YDR427W_folding[c] + YER021W_folding[c] + YFR004W_folding[c] + YFR052W_folding[c] + YOR261C_folding[c] + YPR108W_folding[c] + YKL145W_folding[c] + YDL007W_folding[c] + YDR394W_folding[c] + YOR259C_folding[c] + YOR117W_folding[c] + YGL048C_folding[c] + YHR027C_folding[c] + YIL075C_folding[c] + YHR200W_folding[c] + YLR421C_folding[c] + YER143W_folding[c] + YFR010W_folding[c] + YBR082C_folding[c] + YFL007W_folding[c] + YGL141W_folding[c] + YKL010C_folding[n] + YHL030W_folding[c] -> Mach_proteasome_complex[c]

catalyst: -

COPII transport

ER is the first membrane compartment of the secretory pathway and is where secretory and membrane proteins traffic in the “vesicular” mode first occurs. The exit of correctly folded proteins from ER requires a distinct set of coat proteins and accessory factors. The first step is per-budding complex formation. Sar1, Sec23 and Sec24 are common proteins among different cargos and coat formation mechanisms. Selective export of soluble luminal cargo requires specific cargo receptors. Yeast Erv29 is required for efficiently packaging of the glycosylated alpha-factor pheromone precursor (gpaf) into COPII vesicles and for efficiently secretion of carboxypeptidase Y (CPY)[34]. GPI-anchored proteins require adaptors for cargo selection[35]. A yeast p24 family protein Emp24, which forms a heterotrimeric complex with Erp2, Emp24, and Erv25, has been shown to work as an adaptor for efficient transport of GPI-anchored cargo[36].

Then the budding complex recruits Sec13-Sec31 heterotetramer, providing the outer layer of the coat[37]. Although Sar1, Sec23, Sec24, Sec13 and Sec31 are necessary and sufficient for vesicle formation, additional factors such as Sec16 and Sed4 are also involved in this process[38]. Through interactions with other COPII proteins, Sec16 is thought to facilitate the assembly of

the vesicle coat by stabilizing the pre-budding complex, while Sed4 may regulate the vesicle budding process by inhibiting the GTPase-activating protein (GAP) activity of Sec23.

After budding, vesicles have to move toward the target membrane proceeding through defined and progressive steps: tethering, docking, and fusion. Tethering is mediated by tethering factors such as Uso1[39] and Bug1[40] or multisubunit complexes such as TRAPPI complexes. COPII complex would tether to the cis-Golgi membrane through binding to Sec23 mediated by the TRAPPI complex, including Bet3, Bet5, Trs31, Trs23, Trs33, Trs20, and Trs85[41]. The process of vesicle docking is mediated by another class of proteins named Rabs, which belong to the superfamily of small Ras-like GTPases. Rabs regulate membrane trafficking through interaction with defined effectors. Ypt1 is a Rab protein required for ER to Golgi transport and activated by the TRAPPI complex. Fusion is mediated by SNAREs complex such as Bet1 and Bos1[42].

Here in the model, we divided this process into three steps:

1. COPII pre budding (1A lumen cargo 1B transmembrane cargo 1C GPI anchored cargo)
2. COPII formation
3. COPII tethering docking and fusion

We formulated three different COPII routes for different types of proteins in the model: Transmembrane proteins: coat_trans_membrane (1B); GPI anchored proteins: coat_GPI(1C); Other proteins: coat_other(1A).

Since COPII process has alternatives, we use different proteins to show the example.

Example of COPII_GPI route for protein YBR004C:

```
reaction id:
YBR004C_COPII_GPI_ERGL1C_sec_Sec12p_Sar1p_Sec23p_Sec24p_Emp24p_Erp1p_Erp2p_Erv25p_Bos1p_B
et1p_complex
reaction equation: H2O[er] + GTP[er] + YBR004C_GPI_G6_M8[er] -> H+[er] + phosphate[er] + GDP[er] +
YBR004C_GPI_G6_M8_GPI_G1_COP[er] catalyst:
sec_Sec12p_Sar1p_Sec23p_Sec24p_Emp24p_Erp1p_Erp2p_Erv25p_Bos1p_Bet1p_complex


reaction id: YBR004C_COPII_ERGL_sec_Sec13p_Sec31p_Sec16p_Sed4p_Sec5p_Sec17p_complex
```

reaction equation: YBR004C_GPI_G6_M8_GPI_G1_COP[er] -> YBR004C_GPI_G6_M8_GPI_G2_COP[c]

catalyst: sec_Sec13p_Sec31p_Sec16p_Sed4p_Sec5p_Sec17p_complex

reaction id:

YBR004C_COPII_ERGL_sec_Ypt1p_Uso1p_bug1p_Bet3p_Bet5p_Trs20p_Trs23p_Trs31p_Trs33p_complex

reaction equation: H2O[c] + GTP[c] + YBR004C_GPI_G6_M8_GPI_G2_COP[c] -> H+[c] + phosphate[c] + GDP[c] + YBR004C_GPI_G6_M8[g]

catalyst: sec_Ypt1p_Uso1p_bug1p_Bet3p_Bet5p_Trs20p_Trs23p_Trs31p_Trs33p_complex

Complex formation for enzymes involved in the COPII_GPI:

reaction id:

sec_Sec12p_Sar1p_Sec23p_Sec24p_Emp24p_Erp1p_Erp2p_Erv25p_Bos1p_Bet1p_complex_formation

reaction equation: YNR026C_folding[erm] + 3 YPL218W_folding[erm] + 3 YPR181C_folding[c] + 3 YIL109C_folding[c] + YIL004C_folding[erm] + YGL200C_folding[erm] + YAR002C_A_folding[erm] + YAL007C_folding[erm] + YML012W_folding[erm] + YLR078C_folding[erm] -> sec_Sec12p_Sar1p_Sec23p_Sec24p_Emp24p_Erp1p_Erp2p_Erv25p_Bos1p_Bet1p_complex[erm]

catalyst: -

reaction id: sec_Sec13p_Sec31p_Sec16p_Sed4p_Sec5p_Sec17p_complex_formation

reaction equation: 2 YLR208W_folding[c] + 2 YDL195W_folding[c] + 2 YPL085W_folding[erm] + YCR067C_folding[erm] + YDR166C_folding[c] + YBL050W_folding[v] -> sec_Sec13p_Sec31p_Sec16p_Sed4p_Sec5p_Sec17p_complex[erm]

catalyst: -

reaction id: sec_Ypt1p_Uso1p_bug1p_Bet3p_Bet5p_Trs20p_Trs23p_Trs31p_Trs33p_complex_formation

reaction equation: YFL038C_folding[gm] + 2 YKR068C_folding[g] + YML077W_folding[g] + YDR246W_folding[g] + YDR472W_folding[g] + YDL058W_folding[c] + YDL099W_folding[c] + YBR254C_folding[g] + YOR115C_folding[g] -> sec_Ypt1p_Uso1p_bug1p_Bet3p_Bet5p_Trs20p_Trs23p_Trs31p_Trs33p_complex[gm]

catalyst: -

Example of coat_trans_membrane route for protein YBR021W:

reaction id: YBR021W_COPII_TransM_ERGL1B_sec_Sec12p_Sar1p_Sec23p_Sec24p_Bet1p_Bos1p_complex

reaction equation: H2O[er] + GTP[er] + YBR021W[er] -> H+[er] + phosphate[er] + GDP[er] + YBR021W_COP_coated[er]

catalyst: sec_Sec12p_Sar1p_Sec23p_Sec24p_Bet1p_Bos1p_complex

reaction id: YBR021W_COPII_ERGL_sec_Sec13p_Sec31p_Sec16p_Sed4p_Sec5p_Sec17p_complex

reaction equation: YBR021W_COP_coated[er] -> YBR021W_COP_coated[c]

catalyst: sec_Sec13p_Sec31p_Sec16p_Sed4p_Sec5p_Sec17p_complex

reaction id:

YBR021W_COPII_ERGL_sec_Ypt1p_Uso1p_bug1p_Bet3p_Bet5p_Trs20p_Trs23p_Trs31p_Trs33p_complex

reaction equation: H2O[c] + GTP[c] + YBR021W_COP_coated[c] -> H+[c] + phosphate[c] + GDP[c] + YBR021W[g]

catalyst: sec_Ypt1p_Uso1p_bug1p_Bet3p_Bet5p_Trs20p_Trs23p_Trs31p_Trs33p_complex

Complex formation for enzymes involved in the COPII_TransM:

reaction id: sec_Sec12p_Sar1p_Sec23p_Sec24p_Bet1p_Bos1p_complex_formation

reaction equation: YNR026C_folding[erm] + YPL218W_folding[erm] + YPR181C_folding[c] + YIL109C_folding[c] + YIL004C_folding[erm] + YLR078C_folding[erm] -> sec_Sec12p_Sar1p_Sec23p_Sec24p_Bet1p_Bos1p_complex[erm]

catalyst: -

reaction id: sec_Sec13p_Sec31p_Sec16p_Sed4p_Sec5p_Sec17p_complex_formation

reaction equation: 2 YLR208W_folding[c] + 2 YDL195W_folding[c] + 2 YPL085W_folding[erm] + YCR067C_folding[erm] + YDR166C_folding[c] + YBL050W_folding[v] -> sec_Sec13p_Sec31p_Sec16p_Sed4p_Sec5p_Sec17p_complex[erm]

catalyst: -

reaction id: sec_Ypt1p_Uso1p_bug1p_Bet3p_Bet5p_Trs20p_Trs23p_Trs31p_Trs33p_complex_formation

reaction equation: YFL038C_folding[gm] + 2 YKR068C_folding[g] + YML077W_folding[g] + YDR246W_folding[g] + YDR472W_folding[g] + YDL058W_folding[c] + YDL099W_folding[c] + YBR254C_folding[g] + YOR115C_folding[g] -> sec_Ypt1p_Uso1p_bug1p_Bet3p_Bet5p_Trs20p_Trs23p_Trs31p_Trs33p_complex[gm]

catalyst: -

Example for the COPII_other route for protein YBR092C:

reaction id:

YBR092C_COPII_normal_ERGL1A_sec_Sec12p_Sar1p_Sec23p_Sec24p_Erv29p_Bet1p_Bos1p_complex

reaction equation: H2O[er] + GTP[er] + YBR092C_M8[er] -> H+[er] + phosphate[er] + GDP[er] + YBR092C_M8_COP_coated[er]

catalyst: sec_Sec12p_Sar1p_Sec23p_Sec24p_Erv29p_Bet1p_Bos1p_complex

reaction id: YBR092C_COPII_ERGL_sec_Sec13p_Sec31p_Sec16p_Sed4p_Sec5p_Sec17p_complex
reaction equation: YBR092C_M8_COP_coated[er] -> YBR092C_M8_COP_coated[c]
catalyst: sec_Sec13p_Sec31p_Sec16p_Sed4p_Sec5p_Sec17p_complex

reaction id:
YBR092C_COPII_ERGL_sec_Ypt1p_Uso1p_bug1p_Bet3p_Bet5p_Trs20p_Trs23p_Trs31p_Trs33p_complex
reaction equation: H2O[c] + GTP[c] + YBR092C_M8_COP_coated[c] -> H+[c] + phosphate[c] + GDP[c] + YBR092C_M8[g]
catalyst: sec_Ypt1p_Uso1p_bug1p_Bet3p_Bet5p_Trs20p_Trs23p_Trs31p_Trs33p_complex

Complex formation for enzymes involved in the COPII_other:

reaction id: sec_Sec12p_Sar1p_Sec23p_Sec24p_Erv29p_Bet1p_Bos1p_complex_formation
reaction equation: YNR026C_folding[erm] + 3 YPL218W_folding[erm] + 3 YPR181C_folding[c] + 3 YIL109C_folding[c] + YIL004C_folding[erm] + YLR078C_folding[erm] + YGR284C_folding[erm] -> sec_Sec12p_Sar1p_Sec23p_Sec24p_Erv29p_Bet1p_Bos1p_complex[erm]
catalyst: -

reaction id: sec_Sec13p_Sec31p_Sec16p_Sed4p_Sec5p_Sec17p_complex_formation
reaction equation: 2 YLR208W_folding[c] + 2 YDL195W_folding[c] + 2 YPL085W_folding[erm] + YCR067C_folding[erm] + YDR166C_folding[c] + YBL050W_folding[v] -> sec_Sec13p_Sec31p_Sec16p_Sed4p_Sec5p_Sec17p_complex[erm]
catalyst: -

reaction id: sec_Ypt1p_Uso1p_bug1p_Bet3p_Bet5p_Trs20p_Trs23p_Trs31p_Trs33p_complex_formation
reaction equation: YFL038C_folding[gm] + 2 YKR068C_folding[g] + YML077W_folding[g] + YDR246W_folding[g] + YDR472W_folding[g] + YDL058W_folding[c] + YDL099W_folding[c] + YBR254C_folding[g] + YOR115C_folding[g] -> sec_Ypt1p_Uso1p_bug1p_Bet3p_Bet5p_Trs20p_Trs23p_Trs31p_Trs33p_complex[gm]
catalyst: -

Golgi *N*-glycosylation

N-glycosylated proteins are further modified in Golgi. Upon their arrival, Och1 adds a mannose moiety to the core *N*-linked oligosaccharide[43]. After that, the Golgi *N*-glycosylation modification diverges either to form small core-type oligosaccharides or hyper mannan type[44,45]. Since this kind of data is not available for each protein, we adopted the hyper mannan type in the model, which can be easily modified in the future with available data. As

for the hyper mannan type *N*-glycosylation pathway, a heterodimeric complex M-Pol I, consisting of one copy of Van1 and one copy of Mnn9, is the first enzyme that contributes to the polymerization of mannose in the Golgi[46]. M-Pol II contains five subunits Mnn9, Anp1, Mnn10, Mnn11, and Hoc1, which further elongates the polysaccharide mannan chain[46]. The α-1,6-mannose backbone is further modified with the addition of α-1,2-mannoses by Mnn2 and Mnn5, mannosylphosphate residues by Mnn4p and Mnn6p, and α-1,3-mannoses by Mnn1[47].

We divided this process into four steps in the model:

1. Golgi *N*-glycosylation I with Och1
2. Golgi *N*-glycosylation II with MPOI
3. Golgi *N*-glycosylation II with MPOII
4. Golgi *N*-glycosylation II with Mnn1p Mnn5p and Mnn2p

Example of Golgi *N*-glycosylation for protein YJL139C:

```
reaction id: YJL139C_GLNG_Golgi_N_linked_glycosylation_I_sec_Och1p_complex
reaction equation: 5 GDP-alpha-D-mannose[g] + YJL139C_M8[g] -> 5 GDP[g] + YJL139C_M8_GNG_G1[g]
catalyst: sec_Och1p_complex

reaction id: YJL139C_GLNG_Golgi_N_linked_glycosylation_II_sec_MPOLI_complex
reaction equation: 45 GDP-alpha-D-mannose[g] + YJL139C_M8_GNG_G1[g] -> 45 GDP[g] + YJL139C_M8_GNG_G2[g]
catalyst: sec_MPOLI_complex

reaction id: YJL139C_GLNG_Golgi_N_linked_glycosylation_III_sec_MPoLII_complex
reaction equation: 150 GDP-alpha-D-mannose[g] + YJL139C_M8_GNG_G2[g] -> 150 GDP[g] + YJL139C_M8_GNG_G3[g]
catalyst: sec_MPoLII_complex

reaction id: YJL139C_GLNG_Golgi_N_linked_glycosylation_II_sec_Mnn1p_Mnn2p_Mnn5p_complex
reaction equation: 5 GDP-alpha-D-mannose[g] + YJL139C_M8_GNG_G3[g] -> 5 GDP[g] + YJL139C_M8_GNG_G4[g]
catalyst: sec_Mnn1p_Mnn2p_Mnn5p_complex
```

Complex formation of Golgi *N*-glycosylation involved enzymes:

reaction id: sec_Och1p_complex_formation

reaction equation: YGL038C_folding[erm] -> sec_Och1p_complex[g]

catalyst: -

reaction id: sec_MPOLI_complex_formation

reaction equation: YPL050C_folding[erm] + YML115C_folding[erm] -> sec_MPOLI_complex[gm]

catalyst: -

reaction id: sec_MPoLII_complex_formation

reaction equation: YPL050C_folding[erm] + YEL036C_folding[erm] + YDR245W_folding[erm] + YJL183W_folding[g] + YJR075W_folding[g] -> sec_MPoLII_complex[gm]

catalyst: -

reaction id: sec_Mnn1p_Mnn2p_Mnn5p_complex_formation

reaction equation: YER001W_folding[gm] + YBR015C_folding[gm] + YJL186W_folding[g] -> sec_Mnn1p_Mnn2p_Mnn5p_complex[gm]

catalyst: -

Golgi *O*-glycosylation

In contrast to Golgi *N*-glycosylation, *O*-glycans are synthesized by the stepwise addition of monosaccharides to the first mannose residue added in ER. α-1, 2-mannosyltransferases, Ktr1, Ktr3 and Kre2/Mnt1, participate in the addition of the second mannose residue onto *O*-linked chains in the Golgi[48]. Kre2 has been known to be the primary enzyme responsible for adding the third mannose onto *O*-glycans[48]. Ktr1 and Ktr3 are also able to add mannose, although to a lesser extent than Kre2. The α-1,3- annosyltransferase Mnn1 attaches the fourth mannose residue in the linear chain of up to five mannose residues.

The process was divided into two steps in the model.

1. *O*-glycosylation mannose extension kre2_ktr1_ktr3
2. *O*-glycosylation mannose extension Mnn1

Example for Golgi *O*-glycosylation of YJL137C:

reaction id: YJL137C_GLOG_Golgi_O_linked_manosylation_I_sec_Kre2p_ktr1p_ktr3p_complex

reaction equation: 9 GDP-alpha-D-mannose[g] + YJL137C_OG_M1[g] -> 9 GDP[g] +
YJL137C_OG_M1_GOG_G1[g]
catalyst: sec_Kre2p_ktr1p_ktr3p_complex

reaction id: YJL137C_GLOG_Golgi_O_linked_manosylation_II_sec_Mnn1p_complex
reaction equation: 6 GDP-alpha-D-mannose[g] + YJL137C_OG_M1_GOG_G1[g] -> 6 GDP[g] +
YJL137C_OG_M1_GOG_G2[g]
catalyst: sec_Mnn1p_complex

Complex formation of Golgi *O*-glycosylation involved enzymes:

reaction id: sec_Kre2p_ktr1p_ktr3p_complex_formation
reaction equation: YBR205W_folding[c] + YDR483W_folding[gm] + YOR099W_folding[gm] ->
sec_Kre2p_ktr1p_ktr3p_complex[gm]
catalyst: -

reaction id: sec_Mnn1p_complex_formation
reaction equation: YER001W_folding[gm] ->sec_Mnn1p_complex[gm]
catalyst: -

Mature

We added a mature reaction in the model to indicate the end of the modification.

Example of mature reaction for YJL137C

reaction id: YJL137C_Mature
reaction equation: YJL137C_OG_M1_GOG_G2[g] -> YJL137C_OG_M1_GOG_G2_mature[g]
catalyst: -

Sorting

Mature proteins are transported to their destinations by different vesicle transport.

As for ER or ER membrane proteins, those proteins are transported back to ER via COPI[49]. The COPI complex comprises an ADP-ribosylation factor, Arf1 and the coatomer (Cop1, Sec26, Sec27, Sec21, Ret2, Sec28, and Ret3). The COPI assembly is initiated by the interaction of Arf1 with the Golgi membrane. Arf1 activity is controlled by guanine nucleotide exchange factors (GEF) such as Gea1, Gea2 and Sec7. Once the COPI arrives ER, Arf1 is inactivated to release the cargo proteins. The uncoating and fusion are two separate steps in ER-Golgi vesicle

transport, which were lumped in one reaction in the model for simplicity. By hydrolyzing the ARF1-GTP, the uncoating starts, and then the uncoated vesicle binds to the Golgi membrane by the t-SNAREs[13].

ALP pathway is one of the known trafficking routes from Golgi to Vacuole. Many proteins involved as detecting (Vps1, Swa2), tethering (ClathrinC, Arf1) and docking (t-SNAREC) of the vesicles ALP pathway have been characterized, including Apm3, Apl6, Aps3 and Apl5[50].

The CPY pathway is the default route to the Vacuole from Golgi. A two-step process using AP complexes. The pathway is named after it was suggested to traffic carboxypeptidase Y to the vacuole. AP-1 complex vesicles can transfer proteins from the trans-Golgi to the early or late endosome. After this, the AP-3 complex vesicle moves proteins from the Golgi/endosome to Vacuole[51].

As for proteins located in the cell membrane or extracellular matrix, there are two types of exocytotic vesicles from the trans-Golgi called light density secretory vesicles (LDSV) and heavy density secretory vesicles (HDSV) (upon density-based separation experiments). LDSV is known to carry constitutively expressed cell membrane proteins such as Bgl2, Pma1 and Gas1; and is believed to emerge from the trans-Golgi and transit directly to the cell membrane. HDSV packages soluble, secreted proteins, such as acid phosphates (Pho11, Pho12, Pho5) and invertase (Suc2). Those proteins are usually under transcriptional regulation and induced under certain conditions[52,53].

As for proteins localized in other compartments or membranes, a general reaction is used for the sorting process.

Example of the COPI for protein YJL196C:

```
reaction id:
YJL196C_GLER_COPI_formation_sec_Arf1p_Gea1p_Gea2p_Rer1p_Erd2p_Cop1p_Sec26p_Sec27p_Sec21p_R
et2p_Sec28p_Ret3p_complex
reaction equation: 2 H2O[c] + 2 GTP[c] + YJL196C_mature[g] -> 2 H+[c] + 2 phosphate[c] + 2 GDP[c] +
YJL196C_mature_COPI_G1[c]
```

catalyst:
sec_Arf1p_Gea1p_Gea2p_Rer1p_Erd2p_Cop1p_Sec26p_Sec27p_Sec21p_Ret2p_Sec28p_Ret3p_complex

reaction id:
YJL196C_GLER_COPI_uncoating_and_fission_sec_Rer1p_Ret2p_Cop1p_Sec27p_Sec21p_Bet1p_complex
reaction equation: YJL196C_mature_COPI_G1[c] -> YJL196C_mature[er]
catalyst: sec_Rer1p_Ret2p_Cop1p_Sec27p_Sec21p_Bet1p_complex

Complex formation of COPI involved enzymes:

reaction id:
sec_Arf1p_Gea1p_Gea2p_Rer1p_Erd2p_Cop1p_Sec26p_Sec27p_Sec21p_Ret2p_Sec28p_Ret3p_complex_formation
reaction equation: YDL192W_folding[g] + YJR031C_folding[c] + YEL022W_folding[c] + YDL145C_folding[gm] + YGL137W_folding[g] + YFR051C_folding[g] + YIL076W_folding[g] + YCL001W_folding[gm] + YBL040C_folding[erm] + YDR238C_folding[g] + YNL287W_folding[gm] + YPL010W_folding[g] -> sec_Arf1p_Gea1p_Gea2p_Rer1p_Erd2p_Cop1p_Sec26p_Sec27p_Sec21p_Ret2p_Sec28p_Ret3p_complex[gm]
catalyst: -

reaction id: sec_Rer1p_Ret2p_Cop1p_Sec27p_Sec21p_Bet1p_complex_formation
reaction equation: YIL004C_folding[erm] + YDL145C_folding[gm] + YGL137W_folding[g] + 4 YFR051C_folding[g] + YCL001W_folding[gm] + YNL287W_folding[gm] -> sec_Rer1p_Ret2p_Cop1p_Sec27p_Sec21p_Bet1p_complex[gm]
catalyst: -

Example of the ALP pathway for vacuole membrane protein YJR001W:

reaction id:
YJR001W_ALPtransport_sec_Apl6p_Aps3p_Apm3p_Apl5p_Vam3p_Clc1p_Chc1p_Arf1p_Swa2p_Vps1p_complex
reaction equation: 4 H2O[c] + 4 GTP[c] + YJR001W_mature[g] -> 4 H+[c] + 4 phosphate[c] + 4 GDP[c] + YJR001W_folding[vm]
catalyst: sec_Apl6p_Aps3p_Apm3p_Apl5p_Vam3p_Clc1p_Chc1p_Arf1p_Swa2p_Vps1p_complex

Complex formation of ALP involved enzymes:

reaction id:
sec_Apl6p_Aps3p_Apm3p_Apl5p_Vam3p_Clc1p_Chc1p_Arf1p_Swa2p_Vps1p_complex_formation

reaction equation: YDL192W_folding[g] + YOR106W_folding[vm] + YDR320C_folding[c] + YGR261C_folding[c] + YJL024C_folding[c] + YBR288C_folding[c] + YPL195W_folding[c] + YGR167W_folding[c] + YGL206C_folding[c] + YKR001C_folding[c] -> sec_Apl6p_Aps3p_Apm3p_Apl5p_Vam3p_Clc1p_Chc1p_Arf1p_Swa2p_Vps1p_complex[c]

catalyst: -

Example of the CPY pathway for protein YKL103C:

reaction id: YKL103C_CPYI_sec_Gga1p_Gga2p_Arf1p_Apl4p_Apl2p_Apm1p_Aps1p_Chc1p_Clc1p_Pep12p_Vps45p_Vps5p_Swa2p_complex

reaction equation: 4 H2O[c] + 4 GTP[c] + YKL103C_M8_GNG_G4_mature[g] -> 4 H+[c] + 4 phosphate[c] + 4 GDP[c] + YKL103C_M8_GNG_G4_mature_CPY_G1[v]

catalyst: sec_Gga1p_Gga2p_Arf1p_Apl4p_Apl2p_Apm1p_Aps1p_Chc1p_Clc1p_Pep12p_Vps45p_Vps5p_Swa2p_complex

reaction id: YKL103C_CPYII_sec_Vps4p_Vps27p_Apl6p_Aps3p_Apm3p_Apl5p_Vam3p_complex

reaction equation: H2O[c] + ATP[c] + YKL103C_M8_GNG_G4_mature_CPY_G1[v] -> H+[c] + phosphate[c] + ADP[c] + YKL103C_folding[v]

catalyst: sec_Vps4p_Vps27p_Apl6p_Aps3p_Apm3p_Apl5p_Vam3p_complex

Complex formation of CPY involved enzymes:

reaction id: sec_Gga1p_Gga2p_Arf1p_Apl4p_Apl2p_Apm1p_Aps1p_Chc1p_Clc1p_Pep12p_Vps45p_Vps5p_Swa2p_complex_formation

reaction equation: YDL192W_folding[g] + YDR320C_folding[c] + YGR167W_folding[c] + YGL206C_folding[c] + YDR358W_folding[g] + YHR108W_folding[g] + YPR029C_folding[g] + YKL135C_folding[ce] + YPL259C_folding[c] + YLR170C_folding[c] + YOR036W_folding[v] + YGL095C_folding[c] + YOR069W_folding[gm] -> sec_Gga1p_Gga2p_Arf1p_Apl4p_Apl2p_Apm1p_Aps1p_Chc1p_Clc1p_Pep12p_Vps45p_Vps5p_Swa2p_complex[c]

catalyst: -

reaction id: sec_Vps4p_Vps27p_Apl6p_Aps3p_Apm3p_Apl5p_Vam3p_complex_formation

reaction equation: YOR106W_folding[vm] + YGR261C_folding[c] + YJL024C_folding[c] + YBR288C_folding[c] + YPL195W_folding[c] + YPR173C_folding[erm] + YNR006W_folding[erm] -> sec_Vps4p_Vps27p_Apl6p_Aps3p_Apm3p_Apl5p_Vam3p_complex[c]

catalyst: -

Example of the LDSV for protein YKL217W:

reaction id:
YKL217W_LDSV_sec_Arf1p_Sec3p_Sec5p_Sec6p_Sec8p_Sec10p_Sec15p_Exo70p_Exo84p_Sec4p_Chc1p_Cl
c1p_complex
reaction equation: H2O[c] + GTP[c] + YKL217W_mature[g] -> H+[c] + phosphate[c] + GDP[c] +
YKL217W_folding[ce]
catalyst:
sec_Arf1p_Sec3p_Sec5p_Sec6p_Sec8p_Sec10p_Sec15p_Exo70p_Exo84p_Sec4p_Chc1p_Clc1p_complex

Complex formation of LDSV involved enzymes:

reaction id:
sec_Arf1p_Sec3p_Sec5p_Sec6p_Sec8p_Sec10p_Sec15p_Exo70p_Exo84p_Sec4p_Chc1p_Clc1p_complex_for
mation
reaction equation: YDR166C_folding[c] + YDL192W_folding[g] + YGR167W_folding[c] + YGL206C_folding[c]
+ YER008C_folding[c] + YIL068C_folding[c] + YPR055W_folding[c] + YLR166C_folding[c] +
YGL233W_folding[c] + YJL085W_folding[c] + YBR102C_folding[c] + YFL005W_folding[c] ->
sec_Arf1p_Sec3p_Sec5p_Sec6p_Sec8p_Sec10p_Sec15p_Exo70p_Exo84p_Sec4p_Chc1p_Clc1p_complex[c]
catalyst: -

Example of HDSV for protein YLR155C:

reaction id:
YLR155C_HDSVI_sec_Arf1p_Pep12p_Swa2p_Chc1p_Clc1p_Apl4p_Apl2p_Apm1p_Aps1p_complex
reaction equation: H2O[c] + GTP[c] + YLR155C_M8_GNG_G4_mature[g] -> H+[c] + phosphate[c] + GDP[c] +
YLR155C_M8_GNG_G4_mature[ce]
catalyst: sec_Arf1p_Pep12p_Swa2p_Chc1p_Clc1p_Apl4p_Apl2p_Apm1p_Aps1p_complex

reaction id: YLR155C_HDSVII_sec_Vps1p_Chc1p_Clc1p_complex
reaction equation: H2O[c] + GTP[c] + YLR155C_M8_GNG_G4_mature[ce] -> H+[c] + phosphate[c] + GDP[c] +
YLR155C_folding[e]
catalyst: sec_Vps1p_Chc1p_Clc1p_complex

Complex formation of HDSV involved enzymes:

reaction id: sec_Arf1p_Pep12p_Swa2p_Chc1p_Clc1p_Apl4p_Apl2p_Apm1p_Aps1p_complex_formation

reaction equation: YDL192W_folding[g] + YDR320C_folding[c] + YGR167W_folding[c] + YGL206C_folding[c] + YPR029C_folding[g] + YKL135C_folding[ce] + YPL259C_folding[c] + YLR170C_folding[c] + YOR036W_folding[v] -> sec_Arf1p_Pep12p_Swa2p_Chc1p_Clc1p_Apl4p_Apl2p_Apm1p_Aps1p_complex[c]

catalyst: -

reaction id: sec_Vps1p_Chc1p_Clc1p_complex_formation

reaction equation: YGR167W_folding[c] + YGL206C_folding[c] + YKR001C_folding[c] -> sec_Vps1p_Chc1p_Clc1p_complex[c]

catalyst: -

Example of general pathway for protein YLR240W:

reaction id: YLR240W_transportFromGolgiToOthercompartment

reaction equation: YLR240W_mature[g] -> YLR240W_folding[g]

catalyst: -

Enzyme complex formation

In the model, we formulated reactions to form enzyme complexes, which serve as catalysts for reactions in metabolism and protein modification processes. If this enzyme contains multiple subunits, then the stichometry for the subunits were included in the equation. Stoichiometries for the subunits were collected from the PDB database, available in `Protein_stoichiometry.mat`[54].

Example for enzyme complex formation for secretory machinery complexes has been shown in previous steps. Here we show an example of enzyme complex formation for metabolic reaction r_2141, the complex contains six copies per subunit, thus the stichometry for each subunit is six.

reaction id: r_2141_complex_formation

reaction equation: 6 YKL182W_folding[c] + 6 YPL231W_folding[c] -> r_2141_complex[c]

catalyst: -

Protein processing and complex formation for proteins that are not processed by secretory pathway

Translated nascent peptides need to be folded to form functional complexes for performing specific functions. As for proteins that are not processed in the secretory pathway, we added

a simplified process for folding, misfolding and complex formation, similar to what has been done in proteome-constrained models for *S. cerevisiae*[1] and *Lactococcus lactis*[55]. The folding process in the model is assumed to be in the cytosol and complex formation is assumed in the localization of the complex. As for complexes with multiple subunits, the subunit stoichiometry information collected before was used in the complex formation reaction.

Example of protein processing and complex formation for YBR153W:

```
reaction id: YBR153W_folding_c
reaction equation: YBR153W_peptide[c] -> YBR153W_folding[c]
catalyst: -

reaction id: YBR153W_degradation_misfolding_c
reaction equation: YBR153W_misfolding[c] -> YBR153W_subunit[c]
catalyst: -

reaction id: YBR153W_misfold_c
reaction equation: YBR153W_folding[c] -> YBR153W_misfolding[c]
catalyst: -

reaction id: YBR153W_dilution_misfolding_c
reaction equation: YBR153W_misfolding[c] ->
catalyst: -

reaction id: r_0015_complex_formation
reaction equation: 2 YBR153W_folding[c] -> r_0015_complex[c]
catalyst: -
```

Complex dilution

The complex dilution reactions in the model represent cell division process. Diluted complexes can represent partial protein content in the biomass. Dilution reactions were added for all complexes rather for separate subunits.

Example for complex dilution of r_2141_complex

```
reaction id: r_2141_complex_dilution
reaction equation: r_2141_complex[c] ->
catalyst: -
```

Supplementary Method 5. Turnover rates in the model

Turnover rates for metabolic complexes

The k_{cat} values for metabolic reactions were acquired from the BRENDA database by matching the EC numbers. The k_{cat} extraction process used the criteria as follows[54]. The k_{cat} values for all organisms were downloaded from BRENDA, with only wildtype enzymes, and only the maximal values for the multiple measurements are kept. The assignment of k_{cat} relies on the matching EC number of the subunit and substrate to the dataset. The following criteria was used to determine the k_{cat}:

1. k_{cat} values with both substrate and organism matched in the dataset were prioritized.
2. if there is not such fully matched data, then the median of all k_{cat} values within the organism from the same EC number was used.
3. if only substrate was matched but not organism, then the median of all k_{cat} values with matched substrate from the same EC number was used.
4. if neither organism nor substrate was matched, then the median of all available values with the same EC number was assigned.
5. if no k_{cat} value was available for the EC number, then the k_{cat} value was set with the median of all assigned values.

Note that the k_{cat} value should be adjusted based on the protein stoichiometry information, e.g., the k_{cat} value should multiply 2 for a dimer enzyme, and the median k_{cat} value among subunits was selected for a complex when its subunits had various k_{cat} values. We also manually collect several k_{cat} values, which are available in the GitHub repository: https://github.com/SysBioChalmers/pcSecYeast/tree/main/ComplementaryData/manual_update.xlsx. Besides all these steps, for enzymes with available *in vivo* k_{cat} values (k_{max}), we updated the k_{cat} values in the model to *in vivo* k_{max} since it was demonstrated that utilization of *in vivo* k_{max} could improve the model prediction[56]. Functions: `collectkcats`, `updatekcats` and `matchkappToKcat` were used to collect k_{cat} information and perform changes. Check the corresponding lines in the main function `buildModel` for detailed information. Supplementary Figure 10 describes the kinetic parameter search pipeline.

Turnover rates for secretory complexes

As for k_{cat} parameters for secretory machinery enzymes, proteome abundance data in PaxDb database[57] combining collected PSIM were used to deduct apparent kinetic parameters. The calculation method is based on the machinery abundance and total abundance of proteins processed by the machinery (Supplementary Figure 11). Based on the equation, we can calculate the apparent k_{cat} values for secretory machineries. Protein stoichiometry information is also considered in the calculation. Function: `SimulateSecParam` was used to get the k_{cat} parameters for the secretory machinery complexes.

Turnover rates for translation machinery complexes

Ribosome synthesis rate was collected from the literature, which is 2000/min[58]. Given that the ATP turnover for the proteasome is 110/min (bionumber: 109936)[59], and the ATP cost for eukaryote protein degradation is around 100-200 ATP (bionumber: 112155)[32], and we therefore assumed the proteasome catalytic rate to be 0.5-1 protein/min.

The calculation of ribosomal catalytic rate follows the same method done for *Escherichia coli*[60] and *Lactococcus lactis*[55]. We assumed the equation of ribosomal catalytic rate follow the Michaelis-Menten-type. We collected the mRNA content, protein content and specific growth rates (Supplementary Figure 12)[61–63].

This study[64] showed that there was a linear correlation between the specific growth rate and RNA/protein ratio in *S. cerevisiae.* From the Supplementary Figure 12, we also found clear correlation with the slope being 0.2523 while the intercept 0.1117. The RNA-to-Protein ratio follows the equation.

$$\frac{R}{P} = \frac{\mu}{k_t} + r_0 \quad \mathbf{(1)}$$

in which R is total cellular RNA mass (g/gCDW), P is total cellular protein mass (g/gCDW).

Accordingly, we can estimate k_t = 1/0.2523 = 3.964, while r_0= 0.1117 based on the equation. Ribosomal catalytic rate (aa/ribosome/s) can be formulated as

$$k_{ribo} = \frac{P_s}{n_r} = \frac{\mu P/m_{aa}}{Rf_{rRNA}/m_{rr}} \quad \mathbf{(2)}$$

in which P_s is protein synthesis rate (aa/s), n_r is number of ribosomes, $m_{aa}$is the molecular weight of average amino acid (g/mol), m_{rr} is the mass of rRNA per ribosome (g/mol ribosome),

f_{rRNA} is the fraction of rRNA in total RNA.

Using the R/P equation, then

$$k_{ribo} = \frac{\mu/m_{aa}}{f_{rRNA}/m_{rr}} \cdot \frac{k_t \cdot \mu}{k_t \cdot r_0 + \mu} = \frac{V_{max} \cdot \mu}{\mu + K_m} \quad \mathbf{(3)}$$

in which:

$$V_{max} = \frac{\mu/m_{aa}}{f_{rRNA}/m_{rr}} \cdot k_t \quad \mathbf{(4)}$$

$$K_m = k_t \cdot r_0 \quad \mathbf{(5)}$$

Given that m_{aa} = 109g/mol, m_{rr} = 1.90E6, f_{rRNA}= 0.85 (bionumber: 105192, 100258)[65,66], we can calculate that V_{max} = 22.6, and K_m = 0.443. The ribosomal catalytic rate(aa/ribosome/s) is hence:

$$k_{ribo} = \frac{22.6 \cdot \mu}{\mu + 0.443} \quad \mathbf{(6)}$$

Supplementary Method 6. Constraints in the model

Constraints are required to perform simulations. All constraints were formatted into a linear programming (LP) file for solving as required by the solver SoPlex. Besides the basic flux balanced analysis (FBA) simulation constraint such as:

$$S \cdot V = 0 \;\; (\mathbf{7})$$
$$lb_i \leq V_i \leq \; ub_i \;\; (\mathbf{8})$$

which exist in the basic GEM to represent the steady state and the flux range for each reaction should be between the lower bound and upper bound.

Protein related constraints are added to couple metabolic reactions and the corresponding enzyme enzymes (Supplementary Figure 13), which is represented as the rate of a metabolic reaction is constrained by the concentration of the enzyme that catalyzes it:

$$V_{met,i} \leq k_{cat,i} \cdot [E]_i \;\; (\mathbf{9})$$

where

$$V_{syn} = V_{dil} \; (\mathbf{10})$$
$$V_{dil} = \mu * [E] \; (\mathbf{11})$$
$$[E] = \frac{V_{syn}}{\mu} \; (\mathbf{12})$$

In which, V_{syn} represents the formation rate for one enzyme complex, while V_{dil} represents the dilution rate. Dilution rate of one enzyme complex is coupled to the growth rate, thus we can calculate the enzyme abundance from the synthesis rate and the growth rate (Eq. 12). To be noted here in Equation (12), enzyme complexes are only diluted without degradation in the pcSecYeast, since the degradation happened during the folding process (Supplementary Figure 13). Combining Equation (9-12), we can couple the reaction flux for metabolic reaction with its corresponding enzyme complex formation as:

$$V_{met,i} \leq \frac{k_{cat,i}}{\mu} \cdot V_{syn} \; (\mathbf{13})$$

This type of inequality constraint has been applied to other reactions in protein biosynthesis process, including coupling translation rate and ribosome synthesis rate (Supplementary Figure 13):

$$\sum V_{trans,i} \leq \frac{k_{cat,translate}}{\mu} \cdot V_{syn,ribosome} \quad \textbf{(14)}$$

coupling ribosome synthesis rate and ribosome assembly rate (Supplementary Figure 13), which presents the ribosome synthesis rate is constrained by abundance of ribosome assembly factors:

$$V_{syn,ribosome} \leq \frac{k_{cat,ribo\_assembly}}{\mu} \cdot V_{syn,ribo\_assembly\_factor} \quad \textbf{(15)}$$

coupling protein degradation rate and proteasome synthesis rate (Supplementary Figure 13):

$$\sum V_{subunit\_deg,i} \leq \frac{k_{cat,proteasome}}{\mu} \cdot V_{proteasome} \quad \textbf{(16)}$$

coupling post-translational modification rate with corresponding enzyme synthesis rate (Supplementary Figure 13):

$$\sum V_{\mathrm{sec}\_modification} \leq \frac{k_{cat,i}}{\mu} \cdot V_{\mathrm{syn,sec}\,i} \quad \textbf{(17)}$$

coupling the misfolded protein degradation with the protein translation (Supplementary Figure 13), $\frac{k_{deg,i}}{\mu + k_{deg,i}}$ is collected from the reference. For proteins with no measurement, mean ratio of 30% is used:

$$V_{kdeg,i} \leq \frac{k_{deg,i}}{\mu + k_{deg,i}} \cdot V_{\mathrm{trans,i}} \quad \textbf{(18)}$$

Total proteome is constrained as Equation 19, in which 0.46g is the protein content of 1g biomass.

$$\sum \frac{V_{\mathrm{syn,complex}\,i}}{\mu} + \frac{V_{\mathrm{dummy}}}{\mu} + \frac{V_{\mathrm{dummyER}}}{\mu} + \text{protein content in biomass} = 0.46\text{ g} \quad \textbf{(19)}$$

Besides that, we also included extra constraints in the parameter sensitivity analysis part for CPY accumulation simulation Equation (20-24). Since the protein volume can be roughly considered as the linear correlation with the protein mass[67]. Thus, we can transfer the volume

into simple abundance constraint. Therefore, we calculated the maximum value (0.0786g/gCDW) for total ER proteins from multiple available proteome data for *S. cerevisiae* under diverse conditions[68–73]. Then, we used the value to constrain the ER protein abundance, which is represented as the sum of each ER protein abundance and then converted to the protein synthesis rate according to the Equation 12.

$$\text{ER volume constraint: } \sum \frac{V_{\mathrm{syn,ER,i}}}{\mu} \le 0.0786 \quad (\mathbf{20})$$

The similar constraint was added for ER membrane proteins, ERAD pathway proteins, secretory machinery proteins and retro-translocation enzymes.

$$ER\ membrane\ constraint\text{: } \sum \frac{V_{\mathrm{syn,ERM,i}}}{\mu} \le 0.008 \quad (\mathbf{21})$$

$$ERAD\ constraint\text{: } \sum \frac{V_{\mathrm{syn,ERAD,i}}}{\mu} \le 0.0125 \quad (\mathbf{22})$$

$$Sectory\ machinery\ constraint\text{: } \sum \frac{V_{\mathrm{syn,SEC,i}}}{\mu} \le 0.0244 \quad (\mathbf{23})$$

$$retro - tranlocation\ enzymes\ constraint\text{: } \sum \frac{V_{\mathrm{syn,retro,i}}}{\mu} \le 8.08\text{e-}5 \quad (\mathbf{24})$$

Supplementary references

1. Elsemman, I. E. *et al.* Whole-cell modeling in yeast predicts compartment-specific proteome constraints that drive metabolic strategies. *Nat. Commun.* **13**, 801 (2022).
2. Oftadeh, O. *et al.* A genome-scale metabolic model of *Saccharomyces cerevisiae* that integrates expression constraints and reaction thermodynamics. *Nat. Commun.* **12**, 4790 (2021).
3. Ye, C. *et al.* Comprehensive understanding of *Saccharomyces cerevisiae* phenotypes with whole-cell model WM_S288C. *Biotechnol. Bioeng.* **117**, 1562–1574 (2020).
4. Gutierrez, J. M. *et al.* Genome-scale reconstructions of the mammalian secretory pathway predict metabolic costs and limitations of protein secretion. *Nat. Commun.* **11**, 68 (2020).
5. Irani, Z. A., Kerkhoven, E. J., Shojaosadati, S. A. & Nielsen, J. Genome-scale metabolic model of *Pichia pastoris* with native and humanized glycosylation of recombinant proteins. *Biotechnol. Bioeng.* **113**, 961–969 (2016).
6. The UniProt Consortium. UniProt: the universal protein knowledgebase. *Nucleic Acids Res.* **45**, D158–D169 (2017).
7. Feizi, A., Österlund, T., Petranovic, D., Bordel, S. & Nielsen, J. Genome-Scale Modeling of the Protein Secretory Machinery in Yeast. *PLoS One* **8**, e63284 (2013).
8. Lu, H. *et al.* A consensus *S. cerevisiae* metabolic model Yeast8 and its ecosystem for comprehensively probing cellular metabolism. *Nat. Commun.* **10**, 1–13 (2019).
9. Bosson, R., Guillas, I., Vionnet, C., Roubaty, C. & Conzelmann, A. Incorporation of ceramides into *Saccharomyces cerevisiae* glycosylphosphatidylinositol-anchored proteins can be monitored *in vitro*. *Eukaryot. Cell* **8**, 306–314 (2009).
10. Lopez, S., Rodriguez-Gallardo, S., Sabido-Bozo, S. & Muñiz, M. Endoplasmic Reticulum Export of GPI-Anchored Proteins. *Int. J. Mol. Sci.* **20**, (2019).
11. Dever, T. E. & Green, R. The elongation, termination, and recycling phases of translation in eukaryotes. *Cold Spring Harb. Perspect. Biol.* **4**, 1–16 (2012).
12. de la Cruz, J., Karbstein, K. & Woolford, J. L. Functions of ribosomal proteins in assembly of eukaryotic ribosomes *in vivo*. *Annu. Rev. Biochem.* **84**, 93–129 (2015).
13. Delic, M. *et al.* The secretory pathway: exploring yeast diversity. *FEMS Microbiol. Rev.* **37**, 872–914 (2013).
14. Johnson, N., Powis, K. & High, S. Post-translational translocation into the endoplasmic

reticulum. *Biochim. Biophys. Acta (BBA)-Molecular Cell Res.* **1833**, 2403–2409 (2013).

15. Brown, J. D. *et al.* Subunits of the *Saccharomyces cerevisiae* signal recognition particle required for its functional expression. *EMBO J.* **13**, 4390–4400 (1994).

16. Ogg, S. C., Poritz, M. A. & Walter, P. Signal recognition particle receptor is important for cell growth and protein secretion in *Saccharomyces cerevisiae*. *Mol. Biol. Cell* **3**, 895–911 (1992).

17. Siegel, V. & Walter, P. Each of the activities of signal recognition particle (SRP) is contained within a distinct domain: analysis of biochemical mutants of SRP. *Cell* **52**, 39–49 (1988).

18. Gautschi, M. *et al.* RAC, a stable ribosome-associated complex in yeast formed by the DnaK-DnaJ homologs Ssz1p and zuotin. *Proc. Natl. Acad. Sci. U. S. A.* **98**, 3762–3767 (2001).

19. Young, B. P., Craven, R. A., Reid, P. J., Willer, M. & Stirling, C. J. Sec63p and Kar2p are required for the translocation of SRP-dependent precursors into the yeast endoplasmic reticulum in vivo. *EMBO J.* **20**, 262–271 (2001).

20. Araki, K. & Nagata, K. Protein folding and quality control in the ER. *Cold Spring Harb. Perspect. Biol.* **3**, a007526 (2011).

21. Fujita, M., Umemura, M., Yoko-o, T. & Jigami, Y. *PER1* is required for GPI-phospholipase A2 activity and involved in lipid remodeling of GPI-anchored proteins. *Mol. Biol. Cell* **17**, 5253–5264 (2006).

22. Umemura, M., Fujita, M., Yoko-O, T., Fukamizu, A. & Jigami, Y. *Saccharomyces cerevisiae CWH43* is involved in the remodeling of the lipid moiety of GPI anchors to ceramides. *Mol. Biol. Cell* **18**, 4304–4316 (2007).

23. Kinoshita, T. & Fujita, M. Biosynthesis of GPI-anchored proteins: special emphasis on GPI lipid remodeling. *J. Lipid Res.* **57**, 6–24 (2016).

24. Girrbach, V. & Strahl, S. Members of the evolutionarily conserved PMT family of protein O-mannosyltransferases form distinct protein complexes among themselves. *J. Biol. Chem.* **278**, 12554–12562 (2003).

25. Herscovics, A. Processing glycosidases of *Saccharomyces cerevisiae*. *Biochim. Biophys. Acta* **1426**, 275–285 (1999).

26. Jiang, B. *et al.* *CWH41* encodes a novel endoplasmic reticulum membrane *N*-glycoprotein involved in beta 1,6-glucan assembly. *J. Bacteriol.* **178**, 1162–1171 (1996).

27. Lopata, A., Kniss, A., Löhr, F., Rogov, V. V & Dötsch, V. Ubiquitination in the ERAD Process. *Int. J. Mol. Sci.* **21**, (2020).
28. Paul, P. *et al.* The protein translocation systems in plants - composition and variability on the example of *Solanum lycopersicum*. *BMC Genomics* **14**, 189 (2013).
29. Thibault, G. & Ng, D. T. W. The endoplasmic reticulum-associated degradation pathways of budding yeast. *Cold Spring Harb. Perspect. Biol.* **4**, (2012).
30. Schmidt, C. C., Vasic, V. & Stein, A. Doa10 is a membrane protein retrotranslocase in ER-associated protein degradation. *Elife* **9**, (2020).
31. Jakob, C. A., Burda, P., Roth, J. & Aebi, M. Degradation of misfolded endoplasmic reticulum glycoproteins in *Saccharomyces cerevisiae* is determined by a specific oligosaccharide structure. *J. Cell Biol.* **142**, 1223–1233 (1998).
32. Peth, A., Nathan, J. A. & Goldberg, A. L. The ATP costs and time required to degrade ubiquitinated proteins by the 26 S proteasome. *J. Biol. Chem.* **288**, 29215–29222 (2013).
33. Julius, D., Blair, L., Brake, A., Sprague, G. & Thorner, J. Yeast alpha factor is processed from a larger precursor polypeptide: the essential role of a membrane-bound dipeptidyl aminopeptidase. *Cell* **32**, 839–852 (1983).
34. Caldwell, S. R., Hill, K. J. & Cooper, A. A. Degradation of endoplasmic reticulum (ER) quality control substrates requires transport between the ER and Golgi. *J. Biol. Chem.* **276**, 23296–23303 (2001).
35. Gomez-Navarro, N. & Miller, E. Protein sorting at the ER-Golgi interface. *J. Cell Biol.* **215**, 769–778 (2016).
36. Strating, J. R. P. M. & Martens, G. J. M. The p24 family and selective transport processes at the ER-Golgi interface. *Biol. cell* **101**, 495–509 (2009).
37. Stagg, S. M. *et al.* Structure of the Sec13/31 COPII coat cage. *Nature* **439**, 234–238 (2006).
38. Gimeno, R. E., Espenshade, P. & Kaiser, C. A. *SED4* encodes a yeast endoplasmic reticulum protein that binds Sec16p and participates in vesicle formation. *J. Cell Biol.* **131**, 325–338 (1995).
39. Noda, Y., Yamagishi, T. & Yoda, K. Specific membrane recruitment of Uso1 protein, the essential endoplasmic reticulum-to-Golgi tethering factor in yeast vesicular transport. *J. Cell. Biochem.* **101**, 686–694 (2007).

40. Behnia, R., Barr, F. A., Flanagan, J. J., Barlowe, C. & Munro, S. The yeast orthologue of GRASP65 forms a complex with a coiled-coil protein that contributes to ER to Golgi traffic. *J. Cell Biol.* **176**, 255–261 (2007).

41. Cai, H. *et al.* TRAPPI tethers COPII vesicles by binding the coat subunit Sec23. *Nature* **445**, 941–944 (2007).

42. Lian, J. P. & Ferro-Novick, S. Bos1p, an integral membrane protein of the endoplasmic reticulum to Golgi transport vesicles, is required for their fusion competence. *Cell* **73**, 735–745 (1993).

43. Nakayama, K., Nagasu, T., Shimma, Y., Kuromitsu, J. & Jigami, Y. *OCH1* encodes a novel membrane bound mannosyltransferase: outer chain elongation of asparagine-linked oligosaccharides. *EMBO J.* **11**, 2511–2519 (1992).

44. Munro, S. What can yeast tell us about *N*-linked glycosylation in the Golgi apparatus? *FEBS Lett.* **498**, 223–227 (2001).

45. Conde, R., Cueva, R., Pablo, G., Polaina, J. & Larriba, G. A search for hyperglycosylation signals in yeast glycoproteins. *J. Biol. Chem.* **279**, 43789–43798 (2004).

46. Stolz, J. & Munro, S. The components of the *Saccharomyces cerevisiae* mannosyltransferase complex M-Pol I have distinct functions in mannan synthesis. *J. Biol. Chem.* **277**, 44801–44808 (2002).

47. De Pourcq, K., De Schutter, K. & Callewaert, N. Engineering of glycosylation in yeast and other fungi: current state and perspectives. *Appl. Microbiol. Biotechnol.* **87**, 1617–1631 (2010).

48. Lussier, M., Sdicu, A. M., Bussereau, F., Jacquet, M. & Bussey, H. The Ktr1p, Ktr3p, and Kre2p/Mnt1p mannosyltransferases participate in the elaboration of yeast *O*- and *N*-linked carbohydrate chains. *J. Biol. Chem.* **272**, 15527–15531 (1997).

49. Spang, A. Retrograde traffic from the Golgi to the endoplasmic reticulum. *Cold Spring Harb. Perspect. Biol.* **5**, (2013).

50. Cowles, C. R., Odorizzi, G., Payne, G. S. & Emr, S. D. The AP-3 adaptor complex is essential for cargo-selective transport to the yeast vacuole. *Cell* **91**, 109–118 (1997).

51. Bowers, K. & Stevens, T. H. Protein transport from the late Golgi to the vacuole in the yeast *Saccharomyces cerevisiae*. *Biochim. Biophys. Acta* **1744**, 438–454 (2005).

52. Gurunathan, S., David, D. & JE, G. Dynamin and clathrin are required for the biogenesis of a distinct class of secretory vesicles in yeast. *EMBO J.* **21**, 602–614 (2002).

53. Finger, F. P. & Novick, P. Spatial regulation of exocytosis: lessons from yeast. *J. Cell Biol.* **142**, 609–612 (1998).
54. Chen, Y., Li, F., Mao, J., Chen, Y. & Nielsen, J. Yeast optimizes metal utilization based on metabolic network and enzyme kinetics. *Proc. Natl. Acad. Sci.* **118**, (2021).
55. Chen, Y. *et al.* Proteome constraints reveal targets for improving microbial fitness in nutrient-rich environments. *Mol. Syst. Biol.* **17**, e10093 (2021).
56. Chen, Y. & Nielsen, J. *In vitro* turnover numbers do not reflect *in vivo* activities of yeast enzymes. *Proc. Natl. Acad. Sci. U. S. A.* **118**, (2021).
57. Wang, M., Herrmann, C. J., Simonovic, M., Szklarczyk, D. & von Mering, C. Version 4.0 of PaxDb: Protein abundance data, integrated across model organisms, tissues, and cell-lines. *Proteomics* **15**, 3163–3168 (2015).
58. Huber, A. *et al.* Sch9 regulates ribosome biogenesis via Stb3, Dot6 and Tod6 and the histone deacetylase complex RPD3L. *EMBO J.* **30**, 3052–3064 (2011).
59. Henderson, A., Erales, J., Hoyt, M. A. & Coffino, P. Dependence of proteasome processing rate on substrate unfolding. *J. Biol. Chem.* **286**, 17495–17502 (2011).
60. O'Brien, E. J., Lerman, J. A., Chang, R. L., Hyduke, D. R. & Palsson, B. Ø. Genome-scale models of metabolism and gene expression extend and refine growth phenotype prediction. *Mol. Syst. Biol.* **9**, 693 (2013).
61. Gombert, A. K., Moreira dos Santos, M., Christensen, B. & Nielsen, J. Network identification and flux quantification in the central metabolism of S*accharomyces cerevisiae* under different conditions of glucose repression. *J. Bacteriol.* **183**, 1441–1451 (2001).
62. Ertugay, N. & Hamamci, H. Continuous cultivation of bakers' yeast: change in cell composition at different dilution rates and effect of heat stress on trehalose level. *Folia Microbiol. (Praha).* **42**, 463–467 (1997).
63. Fonseca, G. G., Gombert, A. K., Heinzle, E. & Wittmann, C. Physiology of the yeast *Kluyveromyces marxianus* during batch and chemostat cultures with glucose as the sole carbon source. *FEMS Yeast Res.* **7**, 422–435 (2007).
64. Karpinets, T. V, Greenwood, D. J., Sams, C. E. & Ammons, J. T. RNA:protein ratio of the unicellular organism as a characteristic of phosphorous and nitrogen stoichiometry and of the cellular requirement of ribosomes for protein synthesis. *BMC Biol.* **4**, 30 (2006).

65. von der Haar, T. A quantitative estimation of the global translational activity in logarithmically growing yeast cells. *BMC Syst. Biol.* **2**, 87 (2008).
66. Warner, J. R. The economics of ribosome biosynthesis in yeast. *Trends Biochem. Sci.* **24**, 437–440 (1999).
67. Erickson, H. P. Size and shape of protein molecules at the nanometer level determined by sedimentation, gel filtration, and electron microscopy. *Biol. Proced. Online* **11**, 32–51 (2009).
68. Yu, R., Vorontsov, E., Sihlbom, C. & Nielsen, J. Quantifying absolute gene expression profiles reveals distinct regulation of central carbon metabolism genes in yeast. *Elife* **10**, (2021).
69. Qi, Q. *et al.* Different Routes of Protein Folding Contribute to Improved Protein Production in *Saccharomyces cerevisiae*. *MBio* **11**, (2020).
70. Björkeroth, J. *et al.* Proteome reallocation from amino acid biosynthesis to ribosomes enables yeast to grow faster in rich media. *Proc. Natl. Acad. Sci. U. S. A.* **117**, 21804–21812 (2020).
71. Lahtvee, P.-J. *et al.* Absolute quantification of protein and mRNA abundances demonstrate variability in gene-specific translation efficiency in yeast. *Cell Syst.* **4**, 495–504 (2017).
72. Yu, R. *et al.* Nitrogen limitation reveals large reserves in metabolic and translational capacities of yeast. *Nat. Commun.* **11**, 1881 (2020).
73. Di Bartolomeo, F. *et al.* Absolute yeast mitochondrial proteome quantification reveals trade-off between biosynthesis and energy generation during diauxic shift. *Proc. Natl. Acad. Sci. U. S. A.* **117**, 7524–7535 (2020).
