# Supplementary material for: Improving recombinant protein production by yeast through genome-scale modeling using proteome constraints
Source: Nat Commun. 2022 May 27;13:2969. doi: 10.1038/s41467-022-30689-7 (PMC9142503; doi:10.1038/s41467-022-30689-7)
Supplement: Supplementary file 3 — Description of Additional Supplementary Files [file 41467_2022_30689_MOESM3_ESM.pdf]

File Name: Supplementary Data 1
Description: Extended metabolic reactions for precursor synthesis in the secretory pathway

File Name: Supplementary Data 2
Description: Proteins in the pcSecYeast

File Name: Supplementary Data 3
Description: Reactions in the pcSecYeast

File Name: Supplementary Data 4
Description: Metabolites in the pcSecYeast

File Name: Supplementary Data 5
Description: Direct costs and unit secretory costs for all 497 secretory/cell membrane proteins

File Name: Supplementary Data 6
Description: Protein information for all *S. cerevisiae* proteins

File Name: Supplementary Data 7
Description: Recombinant protein information

File Name: Supplementary Data 8
Description: Predicted results for Insulin precursor

File Name: Supplementary Data 9
Description: Predicted results for α-amylase

File Name: Supplementary Data 10
Description: Predicted results for β-glucosidase

File Name: Supplementary Data 11
Description: Predicted results for Hemoglobin

File Name: Supplementary Data 12
Description: Predicted results for Acid phosphatase

File Name: Supplementary Data 13
Description: Predicted results for Human serum albumin

File Name: Supplementary Data 14
Description: Predicted results for HumanTransferin

File Name: Supplementary Data 15
Description: Predicted results for Human recombinant granulocyte colony stimulating factor

File Name: Supplementary Data 16

Description: Predicted results forInsulin precursor without disulfide bonds

File Name: Supplementary Data 17

Description: Experimental validation results for predicted overexpression targets of α-amylase production

File Name: Supplementary Data 18

Description: Collected mRNA data for α-amylase production strains
