# Supplementary material for: Improving recombinant protein production by yeast through genome-scale modeling using proteome constraints
Source: Nat Commun. 2022 May 27;13:2969. doi: 10.1038/s41467-022-30689-7 (PMC9142503; doi:10.1038/s41467-022-30689-7)
Supplement: Supplementary file 22 — Reporting Summary [file 41467_2022_30689_MOESM22_ESM.pdf]

nature portfolio

Corresponding author(s): Jens Nielsen

Last updated by author(s): 05/05/2022

Reporting Summary

Nature Portfolio wishes to improve the reproducibility of the work that we publish. This form provides structure for consistency and transparency in reporting. For further information on Nature Portfolio policies, see our Editorial Policies and the Editorial Policy Checklist.

Statistics

For all statistical analyses, confirm that the following items are present in the figure legend, table legend, main text, or Methods section.

n/a	Confirmed	
☐	☒	The exact sample size (*n*) for each experimental group/condition, given as a discrete number and unit of measurement
☐	☒	A statement on whether measurements were taken from distinct samples or whether the same sample was measured repeatedly
☐	☒	The statistical test(s) used AND whether they are one- or two-sided<br>*Only common tests should be described solely by name; describe more complex techniques in the Methods section.*
☒	☐	A description of all covariates tested
☒	☐	A description of any assumptions or corrections, such as tests of normality and adjustment for multiple comparisons
☐	☒	A full description of the statistical parameters including central tendency (e.g. means) or other basic estimates (e.g. regression coefficient) AND variation (e.g. standard deviation) or associated estimates of uncertainty (e.g. confidence intervals)
☐	☒	For null hypothesis testing, the test statistic (e.g. *F*, *t*, *r*) with confidence intervals, effect sizes, degrees of freedom and *P* value noted<br>*Give P values as exact values whenever suitable.*
☒	☐	For Bayesian analysis, information on the choice of priors and Markov chain Monte Carlo settings
☒	☐	For hierarchical and complex designs, identification of the appropriate level for tests and full reporting of outcomes
☐	☒	Estimates of effect sizes (e.g. Cohen's *d*, Pearson's *r*), indicating how they were calculated

Our web collection on statistics for biologists contains articles on many of the points above.

Software and code

Policy information about availability of computer code

Data collection

Enzyme turnover numbers (kcat values) were collected from BRENDA database by customized scripts via Application Programming Interface (API).

Data analysis

All code for the analysis and reproducing all figures are included in the GitHub repository:https://github.com/SysBioChalmers/pcSecYeast.
MATLAB (R2019b)
The COBRA toolbox for MATLAB (3.2).
The RAVEN toolbox (2.4.0)
SoPlex (4.0.0)
Gurobi (9.0.1)

DiVenn(2.0) was used for visualization of comparison of predicted targets.

The Protein feature importance analysis uses Python based code, the version for the python packages:
Python (3.7.6)
SHAP (0.39.0)
scikit-learn (0.23.2)
Matplotlib (3.3.2)
pandas (1.1.3)
SciPy (1.5.2)
NumPy (1.20.2)
Detailed instruction about the usage of those toolboxes were documented in the GitHub repository README file. To facilitate further usage, we provide all codes and detailed instruction in GitHub repository: https://github.com/SysBioChalmers/pcSecYeast. All codes to reproduce

figures were also included in the GitHub repository.

For manuscripts utilizing custom algorithms or software that are central to the research but not yet described in published literature, software must be made available to editors and reviewers. We strongly encourage code deposition in a community repository (e.g. GitHub). See the Nature Portfolio guidelines for submitting code & software for further information.

Data

Policy information about availability of data

All manuscripts must include a data availability statement. This statement should provide the following information, where applicable:
- Accession codes, unique identifiers, or web links for publicly available datasets
- A description of any restrictions on data availability
- For clinical datasets or third party data, please ensure that the statement adheres to our policy

Protein Specific Information Matrix (PSIM) information for all proteins in S. cerevisiae was collected from literature and UniProt database. Proteome data used in this study was collected from literature and PaxDb database. Enzyme turnover numbers (kcat values) were collected from BRENDA database. Simulated costs and predicted targets for recombinant protein overproduction are also provided in the Supplementary Data. All data used in this study are included in Supplementary Data and GitHub repository: https://github.com/SysBioChalmers/pcSecYeast. Intermediate results are available in the Zenode: https://doi.org/10.5281/zenodo.6320643. Source data for plotting all figures are provided with this paper.

Field-specific reporting

Please select the one below that is the best fit for your research. If you are not sure, read the appropriate sections before making your selection.

[x] Life sciences [ ] Behavioural & social sciences [ ] Ecological, evolutionary & environmental sciences

For a reference copy of the document with all sections, see nature.com/documents/nr-reporting-summary-flat.pdf

Life sciences study design

All studies must disclose on these points even when the disclosure is negative.

Sample size	No sample size calculation were performed. Three biological replicates were used in the validation of α-amylase overexpression targets, which was chosen following the standard in this field.
Data exclusions	We did not exclude any data.
Replication	All in vivo productions were performed in triplicates and all attempts in the replication were successful. The in silico simulation and analysis were repeated once and results are successfully reproduced.
Randomization	All strains used were randomly picked from the corresponding agar plate. The samples were not randomly allocated into experimental groups because the strains were designed and engineered.
Blinding	Not relevant to this study, as there was no group allocation used.

Reporting for specific materials, systems and methods

We require information from authors about some types of materials, experimental systems and methods used in many studies. Here, indicate whether each material, system or method listed is relevant to your study. If you are not sure if a list item applies to your research, read the appropriate section before selecting a response.

Materials & experimental systems

n/a	Involved in the study	
[x]	[ ]	Antibodies
[x]	[ ]	Eukaryotic cell lines
[x]	[ ]	Palaeontology and archaeology
[x]	[ ]	Animals and other organisms
[x]	[ ]	Human research participants
[x]	[ ]	Clinical data
[x]	[ ]	Dual use research of concern

Methods

n/a	Involved in the study	
[x]	[ ]	ChIP-seq
[x]	[ ]	Flow cytometry
[x]	[ ]	MRI-based neuroimaging
